# Global burden of varicella and herpes zoster across 204 countries, 1990–2021: a temporal trend analysis in the era of the COVID-19 pandemic and projections to 2036

**DOI:** 10.3389/fpubh.2025.1654535

**Published:** 2025-12-05

**Authors:** Zilun Wu, Jinbu Zhang, Jiahao Wei, Chenjin Huang, Weibang Xu, Jiaqiang Wu, Meirong Jiang

**Affiliations:** 1 The First Clinical Medical College, Guangzhou University of Chinese Medicine, Guangzhou, China; 2 The First Affiliated Hospital of Guangzhou University of Chinese Medicine, Guangzhou, China; 3 Guangdong Clinical Research, Academy of Chinese Medicine, Guangdong, China

**Keywords:** varicella and herpes zoster, global burden of disease, sociodemographic index, vaccination, regional disparities, joinpoint regression, post-COVID-19

## Abstract

**Objective:**

To assess the temporal trends and regional disparities of the disease burden of varicella and herpes zoster across 204 countries from 1990 to 2021, analyze the impact of the COVID-19 pandemic era, and project sex-specific incidence trends to 2036.

**Methods:**

Utilizing data from the Global Burden of Disease (GBD) 2021 study, we analyzed the incidence, mortality, and disability-adjusted life years (DALYs) of varicella and herpes zoster. Joinpoint regression analysis identified significant shifts in temporal trends, treating 2019 as a key inflection point to observe the pandemic’s impact. A Bayesian Age-Period-Cohort (BAPC) model was used to project future incidence trends. Analyses were stratified by region, sex, age, and Socio-demographic Index (SDI), and frontier analysis was applied to evaluate the efficiency of health burden management.

**Results:**

From 1990 to 2021, global varicella and herpes zoster cases increased by 19.0% (72.8 to 86.7 million), while age-standardized incidence rates (ASIR) remained stable. Mortality and DALYs declined significantly, with age-standardized mortality rates (ASMR) and DALYs rates (ASDR) decreasing by 50.0 and 36.3%, respectively. A stark disparity remains, with low-SDI regions accounting for over 67% of deaths and DALYs. High-SDI regions, conversely, exhibited a resurgence in varicella and herpes zoster (ASIR: 1,300/100,000) driven by aging populations. The burden followed a U-shaped curve by age, with children <5 years having the highest incidence and adults ≥45 years facing elevated mortality. BAPC modeling projected gradual declines in ASIR for both sexes by 2036.

**Conclusion:**

The dual burdens of varicella and herpes zoster in low-SDI regions and aging-driven herpes zoster resurgence in high-SDI areas necessitate targeted strategies. These include prioritizing pediatric varicella immunization and equitable vaccine distribution in developing nations while enhancing adult herpes zoster booster programs and surveillance in developed ones to meet WHO 2030 targets.

## 1 Background

Varicella and herpes zoster, caused by the varicella-zoster virus (VZV), remain a global public health threat due to high transmissibility and severe complications. Clinically, primary infection causes childhood varicella, after which the virus establishes latency in sensory ganglia and may reactivate decades later as herpes zoster in aging or immunocompromised adults (1). While widespread adoption of vaccines has significantly reduced the disease burden (2), profound regional disparities persist. In resource-constrained settings, insufficient immunization coverage and weak healthcare systems exacerbate transmission, leading to fatal outbreaks, such as one in Pakistan in 2018 (3). Our 2021 GBD analysis shows that 67% of the varicella and herpes zoster burden was concentrated in low-income countries (Table 1).

**Table 1:** Incidence, death, and DALYs of varicella and herpes zoster in 1990 and 2021.

Location	1990		2021		AAPC (95% CI)
**Incidence**	Number (95% UI)	ASR (95% UI)	Number (95% UI)	ASR (95% UI)		
Global	72830736.1 (70112715.1, 75848872.6)	1244.1 (1187.6, 1303.3)	86678086.7 (81687120.9, 92207569.3)	1248.6 (1192.4, 1309.9)	0.012 (0.008–0.016)
Male	36782524.4 (35445267.1, 38284394.7)	1226.8 (1,172, 1283.6)	43,417,799 (41134231, 1.45946087.6)	1,231 (1176.5, 1290.1)	0.011 (0.007–0.015)
Female	36048211.7 (34628333.3, 37581852.4)	1260.3 (1202.1, 1323.5)	43260287.7 (40546110.7, 46,153, 153)	1265.6 (1205.8, 1330.9)	0.013 (0.007–0.018)
Low SDI	10077883.8 (9699953.3, 10412324.9)	1234.8 (1181.2, 1,292)	18357166.2 (17857047.8, 18917274.6)	1233.5 (1179.5, 1290.8)	−0.003 (−0.006–−0.001)
Low-middle SDI	18991791.1 (18233413.1, 19657648.4)	1228.6 (1174.5, 1285.3)	23,085,939 (22145458.1, 24089307.5)	1228.6 (1175.3, 1284.3)	0.000 (−0.001–0.002)
Middle SDI	23223118.9 (22363326.8, 24173597.6)	1250.1 (1188.9, 1313.3)	24399358.7 (22686940.3, 26291839.3)	1242.4 (1183.7, 1302.7)	−0.020 (−0.030–−0.010)
High-middle SDI	11511421.8 (10928050.6, 12142526.9)	1218.3 (1165.1, 1274.9)	10959062.3 (9907928.7, 12071150.8)	1229.2 (1173.9, 1289.4)	0.030 (0.024–0.036)
High SDI	8966773.8 (8,378,302, 9585144.3)	1272.3 (1214.5, 1336.4)	9810608.6 (8809926.8, 10914845.5)	1275.3 (1211.3, 1346.2)	0.006 (−0.020–0.032)
Andean Latin America	588259.4 (569,113, 608264.5)	1,217 (1164.9, 1275.6)	751375.5 (717062.7, 789686.3)	1214.4 (1,162, 1271.4)	−0.007 (−0.009 - − 0.005)
Australasia	177735.2 (175067.1, 182,319)	1097.8 (1075.6, 1133.7)	252279.1 (236248.6, 269767.5)	1155.1 (1111.3, 1204.9)	0.164 (0.152–−0.177)
Caribbean	481459.5 (464769.3, 498,841)	1222.9 (1171.6, 1279.9)	503290.8 (476527.2, 532,566.3)	1220.9 (1170.2, 1277.3)	−0.005 (−0.007–00.003)
Central Asia	1018570.9 (987376.6, 1057861.6)	1143.4 (1106.3, 1187.8)	1122690.9 (1085183.1, 1168623.3)	1142.6 (1104.6, 1188.2)	−0.002 (−0.003–−0.002)
Central Europe	1106627.1 (1056003.1, 1163711.4)	1137.9 (1099.1, 1184.7)	790081.9 (733,871, 853426.5)	1138.4 (1098.3, 1185.7)	0.002 (−0.003–0.007)
Central Latin America	2597009.3 (2524673.4, 2671427.5)	1227.7 (1,178, 1285.4)	2550407.7 (2415808.4, 2702359.7)	1,225 (1174.3, 1280.7)	−0.007 (−0.009–00.005)
Central Sub-Saharan Africa	1165948.5 (1125495.9, 1203486.9)	1227.3 (1175.3, 1286.8)	2295733.9 (2237814.8, 2,370,006)	1224.8 (1170.9, 1284.4)	−0.006 (−0.008–−0.005)
East Asia	14405609.2 (13670477.6, 15167588.4)	1276.4 (1212.7, 1346.8)	12955288.9 (11502811.5, 14496906.7)	1,272 (1209.5, 1341.1)	−0.012 (−0.018–00.006)
Eastern Europe	2,048,252 (1930371.5, 2171857.6)	1146.6 (1109.2, 1191.5)	1425011.4 (1312769.4, 1546521.2)	1145.5 (1107.5, 1191.4)	−0.003 (−0.004–00.002)
Eastern Sub-Saharan Africa	4037661.1 (3897736.3, 4,162,191)	1228.9 (1175.8, 1287.6)	7009730.9 (6826524.5, 7,221,630.8)	1227.6 (1,173, 1285.6)	−0.004 (−0.004–00.003)
High-income Asia Pacific	1670224.1 (1,528,255,1842925.3)	1311.6 (1242.9,1388.9)	1674998.7 (1456357.5,1932710.7)	1333.1 (1256.9,1414.3)	0.055 (0.032–0.079)
High-income North America	3034489.3 (2849970.1, 3,235,810)	1264.2 (1205.5, 1330.2)	3427339.9 (3,111,166, 3782,240.5)	1257.3 (1195.8, 1326.6)	−0.029 (−0.119–0.061)
North Africa and Middle East	5,591,751 (5408366.8, 5820810.3)	1224.4 (1168.3, 1284.9)	7346951.7 (7019457.6, 7709547.4)	1225.6 (1,170, 1286.7)	0.003 (0.001–0.005)
Oceania	110593.2 (105545.9, 115255.8)	1283.4 (1217.7, 1,354)	219331.6 (209162.1, 229577.9)	1285.1 (1218.8, 1356.9)	0.004 (0.004–0.005)
South Asia	17108617.8 (16421476.4, 17,688,471)	1217.1 (1166.2, 1275.3)	19878759.2 (18969596.9, 20916085.2)	1210.6 (1156.3, 1267.3)	−0.016 (−0.018–00.014)
Southeast Asia	6559469.6 (6207764.5, 6,864,616)	1255.9 (1180.9, 1326.5)	7791214.1 (7309398.3, 8311664.3)	1253.2 (1,178, 1324.9)	−0.007 (−0.010–00.005)
Southern Latin America	637560.7 (608224.1, 670483.5)	1265.8 (1207.3, 1334.5)	619146.5 (568877.4, 675,047)	1271.6 (1212.1, 1339.5)	0.015 (0.013–0.018)
Southern Sub-Saharan Africa	832333.9 (809243.3, 860,463)	1229.5 (1176.4, 1288.3)	957804.3 (919,140, 1000782.1)	1230.3 (1177.4, 1,289)	0.002 (0.001–0.003)
Tropical Latin America	1935282.7 (1866269.8, 2011850.7)	1229.5 (1178.2, 1288.6)	2284188.2 (2150671.9, 2439423.4)	1229.6 (1177.6, 1,289)	0.000 (−0.002–0.002)
Western Europe	3680450.3 (3395437.7, 3997080.7)	1284.2 (1226.4, 1348.4)	4043391.4 (3620575.4, 4512834.7)	1295.3 (1228.3, 1367.1)	0.023 (0.001–0.045)
Western Sub-Saharan Africa	4,042,831 (3895022.6, 4175407.4)	1232.1 (1178.8, 1290.7)	8,779,070 (8541948.5, 9032616.9)	1229.5 (1176.1, 1287.7)	−0.007 (−0.008–−0.005)
Death					
Global	15632.9 (14140.8, 17384.6)	0.4 (0.3, 0.4)	13930.7 (12584.9, 15,605)	0.2 (0.2, 0.2)	−1.95 (−2.02–−1.87)
Male	7699.6 (6919.8, 8521.9)	0.4 (0.3, 0.4)	6486.2 (5728.5, 7314.8)	0.2 (0.2, 0.2)	−2.10 (−2.18–−2.03)
Female	7933.3 (7074.8, 8912.4)	0.3 (0.3, 0.4)	7444.5 (6614.5, 8337.1)	0.2 (0.2, 0.2)	−1.85 (−1.94–−1.76)
Low SDI	3799.7 (3169.1, 4608.2)	1.2 (1,1.3)	4582.9 (3734.3, 5457.5)	0.7 (0.6, 0.8)	−1.65 (−1.78–−1.53)
Low-middle SDI	5334.2 (4663.3, 6069.1)	0.7 (0.6, 0.8)	4786.2 (4250.1, 5381.5)	0.4 (0.3, 0.4)	−1.89 (−2.16–−1.61)
Middle SDI	4249.1 (3927.9, 4595.2)	0.4 (0.4, 0.4)	2363.5 (2149.9, 2573.4)	0.1 (0.1, 0.1)	−3.89 (−3.96–−3.82)
High-middle SDI	1301.7 (1168.8, 1444.3)	0.2 (0.1, 0.2)	543.6 (481.6, 606.5)	0 (0, 0)	−4.28 (−4.42–−4.15)
High SDI	937 (842.1, 1,004)	0.1 (0.1, 0.1)	1643.1 (1329.6, 1845.1)	0.1 (0.1, 0.1)	−1.19 (−1.43–−0.95)
Andean Latin America	148.9 (120.4, 183.2)	0.6 (0.4, 0.7)	82 (63.1, 106)	0.1 (0.1, 0.2)	−4.32 (−4.84–−3.81)
Australasia	13.5 (11.8, 15.3)	0.1 (0.1, 0.1)	76 (60.9, 89.2)	0.1 (0.1, 0.1)	1.98 (1.03–2.94)
Caribbean	131.4 (100.6, 164.8)	0.4 (0.3, 0.5)	124.7 (95, 165.2)	0.3 (0.2, 0.4)	−1.40 (−1.85–−0.95)
Central Asia	51.4 (42.1, 61.6)	0.1 (0.1, 0.1)	32.9 (25.7, 41.4)	0 (0, 0.1)	−2.92 (−3.35–−2.48)
Central Europe	61.8 (55.6, 68.8)	0.1 (0.1, 0.1)	23.6 (20.1, 27.4)	0 (0, 0)	−4.87 (−5.28–−4.45)
Central Latin America	402.7 (376, 430.9)	0.3 (0.3, 0.3)	231.7 (198.4, 273.9)	0.1 (0.1, 0.1)	−3.44 (−3.78–−3.09)
Central Sub-Saharan Africa	412.7 (307.6, 549.4)	1.4 (1, 1.8)	497.7 (364.6, 651.3)	0.8 (0.5, 1)	−1.92 (−2.14–−1.70)
East Asia	2733.3 (2453.2, 3,025)	0.4 (0.3, 0.4)	699.9 (594.4, 809.4)	0 (0, 0.1)	−6.68 (−6.84–−6.52)
Eastern Europe	52.3 (48.8, 56.2)	0 (0, 0)	44.1 (39.1, 48.9)	0 (0, 0)	0.27 (−0.18–0.73)
Eastern Sub-Saharan Africa	1583.3 (1,274, 2010.8)	1.3 (1.1, 1.5)	1766.3 (1432.7, 2186.8)	0.7 (0.6, 0.8)	−2.00 (−2.17–−1.83)
High-income Asia Pacific	94.4 (81, 109.6)	0.1 (0.1, 0.1)	148.8 (108.8, 175.3)	0 (0, 0)	−3.32 (−3.68–−2.95)
High-income North America	250.6 (223.5, 269.9)	0.1 (0.1, 0.1)	487.7 (404.7, 543.2)	0.1 (0.1, 0.1)	−0.12 (−0.50–0.27)
North Africa and Middle East	1052.2 (904.2, 1231.7)	0.5 (0.4, 0.6)	669.7 (548.4, 799)	0.2 (0.1, 0.2)	−3.77 (−3.98 –−3.56)
Oceania	24.7 (17.8, 33.6)	0.5 (0.4, 0.6)	47.1 (34.2, 64.1)	0.6 (0.4, 0.7)	−0.92 (−1.13–−0.72)
South Asia	4857.1 (4196.5, 5608.7)	0.7 (0.6, 0.9)	4497.2 (4040.6, 5035.7)	0.4 (0.3, 0.4)	−1.79 (−2.13–−1.45)
Southeast Asia	1436.2 (1269.3, 1624.6)	0.5 (0.4, 0.6)	1173.9 (1036.8, 1328.3)	0.2 (0.2, 0.3)	−2.44 (−2.55–−2.33)
Southern Latin America	41.8 (36.4, 47.7)	0.1 (0.1, 0.1)	29.9 (25.4, 35.2)	0 (0, 0.1)	−1.94 (−2.80–−1.07)
Southern Sub-Saharan Africa	167 (144.3, 192)	0.5 (0.4, 0.6)	177.1 (147.3, 213.5)	0.3 (0.3, 0.4)	−1.52 (−1.84–−1.20)
Tropical Latin America	235.4 (205.7, 272.5)	0.2 (0.1, 0.2)	227.2 (196.4, 255.6)	0.1 (0.1, 0.1)	−1.39 (−1.93–−0.86)
Western Europe	514.1 (449.4, 558.5)	0.1 (0.1, 0.1)	959.7 (765.8, 1089.2)	0.1 (0.1, 0.1)	−0.84 (−1.04–−0.65)
Western Sub-Saharan Africa	1368.1 (1149.5, 1623.6)	1.1 (1, 1.2)	1933.4 (1459.2,2402.6)	0.7 (0.6,0.7)	−1.70 (−1.87–−1.52)
DALYs					
Global	1065063.4 (936651.1, 1230332.7)	19.3 (17, 22.1)	886066.5 (744313.3, 1060071.5)	12.3 (10.4, 14.7)	−1.42 (−1.49–−1.35)
Male	541091.2 (473499.4, 621411.1)	19.6 (17.3, 22.3)	437069.4 (364592.2, 529224.3)	12.2 (10.2, 14.8)	−1.50 (−1.58–−1.42)
Female	523972.2 (455932.1, 610006.4)	19.1 (16.7, 22.1)	448997.2 (376428.2, 535,953)	12.4 (10.5, 14.7)	−1.35 (−1.44–−1.27)
Low SDI	267978.1 (217226.6, 336401.4)	42.4 (36.4, 50)	315456.8 (245813.6, 386443.6)	26.2 (21.6, 31.3)	−1.53 (−1.72–−1.34)
Low-middle SDI	358324.1 (304879.7, 418560.6)	27.7 (24.3, 31.6)	277016.9 (237405.5, 324171.5)	16 (13.8, 18.5)	−1.70 (−1.93–−1.47)
Middle SDI	286866.6 (257773.2, 319771.2)	17.6 (15.9, 19.7)	165022.6 (132320.1, 209871.3)	7.5 (6.1, 9.4)	−2.72 (−2.81–−2.63)
High-middle SDI	96488.5 (82909.4, 112521.1)	10.1 (8.7, 11.7)	59604.5 (41731.2, 82,261)	4.5 (3.3, 5.9)	−2.60 (−2.71–−2.50)
High SDI	54633.3 (42683.8, 70529.6)	6.2 (5, 7.8)	68219.8 (49838.9, 92850.1)	4.7 (3.4, 6.3)	−0.92 (−1.04–−0.80)
Andean Latin America	8961.6 (6881.5, 11558.7)	21.8 (17.8, 26.9)	4,651 (3601.3, 5986.9)	7.5 (5.9, 9.6)	−3.45 (−3.82–−3.07)
Australasia	642 (518.3, 791)	3.2 (2.7, 4)	1829.5 (1465.5, 2282.1)	4.5 (3.6, 5.7)	1.06 (0.63–1.48)
Caribbean	8917.5 (6505.3, 11690.7)	23.8 (18, 30.7)	7886.9 (5527.7, 10710.5)	18.6 (12.7, 25.8)	−0.83 (−1.31–−0.35)
Central Asia	3656.9 (2927.4, 4400.6)	5.1 (4.2, 6.2)	3412.6 (2500.6, 4546.7)	3.7 (2.7, 4.8)	−1.04 (−1.22–−0.87)
Central Europe	4733.9 (3764.3, 5953.3)	4.2 (3.5, 5.1)	2980.8 (1957.8, 4359.2)	2.1 (1.4, 3)	−2.20 (−2.43–−1.97)
Central Latin America	30661.6 (28155.2, 33455.4)	16.6 (15.1, 18.3)	16506.5 (13380.4, 20711.5)	7 (5.7, 8.7)	−2.78 (−3.09–−2.47)
Central Sub-Saharan Africa	30173.4 (20623.2, 41315.9)	44.2 (35.2, 56.2)	34473.3 (23821.4, 47591.1)	25 (18.8, 32.1)	−1.85 (−2.07–−1.62)
East Asia	181, 237 (156133.2, 208894.7)	17.5 (15.2, 20)	75023.3 (50886.3, 105002.2)	4.7 (3.4, 6.4)	−4.16 (−4.32–−4.00)
Eastern Europe	8455.9 (6696.5, 10684.7)	4.3 (3.6, 5.3)	7,307 (5477.7, 9,760)	4.4 (3.7, 5.4)	0.05 (−0.27–0.37)
Eastern Sub-Saharan Africa	114969.5 (90002.5, 151658.5)	46.5 (38.6, 56.9)	125412.8 (96009.9, 159131.4)	27.2 (22.2, 32.7)	−1.69 (−1.85–−1.53)
High-income Asia Pacific	10,014 (7207.4, 13627.6)	6.2 (4.7, 8.2)	11822.6 (7,555, 17170.6)	4.3 (2.8,6.3)	−1.15 (−1.29–−1.00)
High-income North America	16661.6 (13077.7, 21659.5)	5.7 (4.6, 7.3)	22673.5 (16803.1, 30403.9)	4.9 (3.7, 6.5)	−0.49 (−0.70–−0.28)
North Africa and Middle East	70586.8 (58901.6, 87156.3)	19.4 (16.8, 22.8)	50223.2 (39959.5, 62108.4)	8.9 (7.2, 11)	−2.47 (−2.63–−2.31)
Oceania	1794.9 (1259.1, 2510.1)	26.3 (20, 34.3)	3351.5 (2345.5, 4675.8)	23 (17.4, 30.2)	−0.44 (−0.69–−0.18)
South Asia	326560.5 (273559.4, 387, 295)	27.7 (24, 31.6)	245497.3 (211023.2, 291636.6)	15.8 (13.7, 18.6)	−1.75 (−1.94–−1.56)
Southeast Asia	94346.5 (81472.9, 110874.3)	20.9 (18.3, 24)	71329.1 (59072.7, 86809.6)	11.8 (9.9, 14.2)	−1.82 (−1.91–−1.74)
Southern Latin America	4378.1 (3,689, 5334.5)	8.8 (7.4, 10.7)	3,599 (2716.3, 4777.4)	5.8 (4.5, 7.4)	−1.40 (−1.87–−0.92)
Southern Sub-Saharan Africa	10,372 (8738.2, 12347.2)	19 (16.5, 21.8)	11535.4 (9270.9, 14703.5)	15.7 (12.8, 19.7)	−0.69 (−0.96–−0.41)
Tropical Latin America	21438.1 (18501.3, 25220.1)	13.8 (11.9, 16.2)	16981.2 (13879.2, 21001.9)	8.2 (6.7, 10)	−1.68 (−2.13–−1.24)
Western Europe	24061.8 (18250.5, 31628.8)	5.8 (4.5, 7.4)	31120.5 (23441.3, 41365.7)	4.8 (3.5, 6.5)	−0.59 (−0.67–−0.51)
Western Sub-Saharan Africa	92439.5 (73,977, 115425.9)	37.5 (32.1, 43.9)	138449.7 (100313.3, 177945.3)	24.4 (19, 29.9)	−1.37 (−1.48–−1.27)

AAPC (95% CI): average annual percentage change (95% confidence interval) ASR (95% UI): age-standardized rate (95% uncertainty interval) number (95% UI): estimated number (95% uncertainty interval) DALYs: disability-adjusted life years.

Most prior studies have focused on single countries, leaving gaps in systematic analyses of long-term global burden trends, Socio-demographic Index (SDI)-driven health inequities, and age-sex-specific risks (4). Furthermore, deep analyses of regional heterogeneity and social determinants are lacking (5), and the long-term impacts of the COVID-19 pandemic on immunization programs remain underquantified.

Varicella vaccines were first approved for healthy children in Japan and South Korea in 1989 and introduced to the U.S. market in 1995. To date, 29 countries have included varicella vaccines in their national immunization programs (UIPs), though Asian nations like China and India have not yet done so. However, with the expanding spectrum of VZV-related diseases and growing evidence for co-introducing varicella and herpes zoster vaccines in routine immunization, this status quo is under renewed scrutiny (6). The pandemic significantly shifted immunization priorities. A September 2024 WHO and UNICEF report highlighted that declines in routine vaccination (7) increased the risk of resurgences. For example, the coverage rate of the varicella vaccine has decreased significantly in Italy (8, 9), and Brazil experienced surging outbreaks (10). This further widens the gap: high-income countries not only have better immunization programs, but also can reduce risks by administering booster doses of the shingles vaccine to adults. However, low- and middle-income countries still face challenges in terms of vaccine access and public awareness (11).

Using GBD 2021 data, this study aims to: (1) Quantify spatiotemporal trends in the VZV burden from 1990–2021; (2) Analyze regional disparities across SDI strata; (3) Evaluate associations between vaccine coverage and disease burden; and (4) Project the pandemic’s long-term effects on incidence through 2036. These findings will provide empirical evidence for WHO’s Immunization Agenda 2030.

## 2 Methods

### 2.1 Data sources

The 2021 Global Burden of Disease (GBD) study synthesizes epidemiological data from 204 countries and territories to assess the burden of varicella and herpes zoster.^1^ This comprehensive dataset employs standardized methods to quantify health losses associated with 369 diseases, injuries, and impairments (12), as well as 88 risk factors, ensuring cross-regional comparability.

The GBD reporting system is based on a systematic synthesis of multiple data sources, including national surveillance systems, hospital discharge records (using ICD-9 and ICD-10 codes), vital registration data, verbal autopsies, censuses, household surveys, specific disease registries, and published epidemiological literature. For this study, the GBD 2021 diagnostic criteria for primary varicella (chickenpox) and herpes zoster (shingles) are primarily derived from clinical diagnosis. It is important to note that in many regions, particularly those with a low Socio-demographic Index (SDI), case confirmation often relies on clinical diagnosis without routine laboratory confirmation.

The GBD framework employs advanced statistical techniques to address missing data and reporting inconsistencies, particularly in conflict-affected and surveillance-limited regions (13, 14). The framework applies Cause of Death Ensemble modeling (CODEm) and Bayesian meta-regression (DisMod-MR 2.1) for data harmonization, while spatiotemporal Gaussian process regression leverages data from neighboring regions to estimate burden in data-sparse areas. Population surveys and satellite data supplement conflict zone demographics. Through 1,000 bootstrap iterations, the model generates uncertainty intervals (UIs) to quantify estimation precision, with cross-validation ensuring robustness in low-data environments (15). This comprehensive approach systematically adjusts for demographic biases and reporting variations, providing reliable burden estimates where health infrastructure is weakest, though it is subject to the limitations of the underlying primary data. All analyses adhere to the Guidelines for Accurate and Transparent Health Estimates Reporting (GATHER) (16).

Other data can be accessed through the online query tools available on both the Institute for Health Metrics and Evaluation (IHME) website^2^ and the World Health Organization (WHO) Global Health Observatory (GHO) portal.^3^ Detailed information on the original data sources for varicella and herpes zoster, including provider, collection method, and sample size, is available via the IHME’s data transparency website.^4^ No permission was required for retrieval.

### 2.2 Socio-demographic index (SDI)

The Socio-demographic Index (SDI) quantifies national development (0–1 scale) using three components: lag-distributed income, educational attainment, and total fertility rate (17). Countries were classified into quintiles (low-high SDI) to analyze varicella and herpes zoster burden disparities, as this approach: (1) enables cross-country comparisons, (2) captures epidemiological transition patterns, and (3) maintains GBD methodological consistency (18). Smooth spline models assessed nonlinear relationships between SDI and age-standardized incidence/mortality/DALY rates across 21 regions. The quintile stratification reflects meaningful development thresholds while ensuring sufficient sample sizes for robust analysis. This standardized classification system allows for effective comparison of disease burden across different socioeconomic contexts, with detailed methodology available in prior GBD publications (17).

### 2.3 DisMod-MR2.1

DisMod-MR 2.1 (19), the Bayesian meta-regression tool developed by IHME for GBD studies, employs a compartmental modeling framework to synthesize heterogeneous epidemiological data. The model’s hierarchical structure (global-super region-region-country-subnational) enables cross-scale information borrowing, particularly valuable for handling zero-count scenarios in high-SDI regions. These features allow robust burden estimation even when observed counts are zero, by incorporating information from demographically similar populations and historical trends. The differential equation system simultaneously models incidence, prevalence, and mortality while accounting for age-sex interactions and healthcare access gradients characteristic of high-SDI settings.

### 2.4 Burden metrics

Age-standardized incidence rates (ASIR), mortality rates (ASMR), and DALY rates (ASMR) per 100,000 population were calculated using the GBD reference population structure. Uncertainty intervals (95% UI) were derived from 1,000 Monte Carlo simulations (20, 21), incorporating input data variability, measurement error corrections, and residual non-sampling error estimates. The joinpoint regression model, a linear statistical model, was employed to evaluate temporal trends in disease burden. This model uses least squares estimation to identify changes in incidence rates, addressing the subjectivity inherent in traditional linear trend analyses. Uncertainty intervals (UIs) were calculated as the 2.5th to 97.5th percentile range of 1,000 bootstrap iterations. For regions with complete data, UIs were derived from the GBD database’s pre-computed bounds (upper/lower values). In data-sparse areas, DisMod-MR 2.1 supplemented estimates through: (1) spatial smoothing across neighboring regions, (2) borrowing strength from demographically similar populations, and (3) temporal trend extrapolation. Graphical UIs (shaded bands) were generated using validated R package algorithms (quantile regression with smoothing splines). These implementations have undergone rigorous statistical validation and are suitable for most analytical scenarios, ensuring robust uncertainty characterization.

### 2.5 Temporal trends

Joinpoint regression (Joinpoint Software v4.9.1.0) was selected over conventional trend analyses for three key reasons: (1) Its segmented modeling approach identifies inflection points where disease burden trends significantly change direction, critical for capturing policy impacts or epidemiological shifts in GBD studies; (2) The method’s piecewise linear regression reduces autocorrelation artifacts inherent in time-series data, enhancing result reliability; and (3) Permutation testing (*α* = 0.05) objectively determines optimal joinpoint numbers while controlling overfitting. We quantified trends using average annual percentage change (AAPC) with 95% Cis (22, 23) and annual Percent Change (APC), testing against zero-change baselines. This approach optimally balances sensitivity to abrupt transitions (e.g., pandemic effects) with statistical rigor, particularly for heterogeneous global data where uniform trends are improbable.

### 2.6 Age- and sex-stratified analysis

Age-specific incidence, mortality, and DALYs were disaggregated by sex and nearly 5-year age cohorts (24). Rate ratios (male-to-female) with 95% UIs were computed to quantify gender disparities.

### 2.7 Assess the potential impact of the COVID-19 pandemic

To assess the potential impact of the COVID-19 pandemic, this study specifically focused on trend inflection points occurring in or after 2019 within the Joinpoint regression analysis (analyzing the 2016–2021 period). It is crucial to clarify that this method does not incorporate ‘COVID-19 risk factors’ as direct variables into the burden estimation models. Rather, it statistically identifies significant shifts in the temporal trends of the VZV disease burden. Any such changes observed post-2019 are subsequently interpreted in the discussion section in the context of external evidence, such as disruptions to routine immunization programs and altered healthcare-seeking behaviors.

### 2.8 Decomposition analysis

This study quantified drivers of global VZV disease-related DALYs changes (1990–2021) through demographic decomposition analysis, distinguishing three components: (1) the epidemiological effect (r_effect) reflecting incidence/mortality changes from vaccination and healthcare interventions while controlling for demographic factors; (2) the population growth effect (p_effect) measuring DALYs variation due to population size changes with constant age structure and disease risks; and (3) the age-structural effect (a_effect) assessing impacts of shifting age distributions while holding population size and epidemiology constant. The total change (ΔDALYs) represents the sum of these independently calculated effects (ΔDALYs = r_effect + p_effect + a_effect), the statistical values of the decomposition analysis have been uploaded to Sheet3 of Excel.

### 2.9 Bayesian age-period-cohort (BAPC) model projections

We employed a Bayesian Age-Period-Cohort (BAPC) model to characterize and project the incidence trends of varicella and herpes zoster among males and females up to 2030. This advanced model integrated GBD incidence data (1990–2021) and population demographics with IHME population projections (2022–2036) to construct a Bayesian framework-based age-period-cohort model, utilizing the Integrated Nested Laplace Approximation (INLA) algorithm for parameter estimation. Reporting of this work adheres to the Strengthening the Reporting of Cohort, Cross-Sectional, and Case–Control Studies (STROCSS) criteria (25). Statistical analyses were performed using R version 4.2.3, providing a scientific basis for disease burden assessment and public health decision-making. The Bayesian framework is particularly suited for robust predictions with small sample sizes.

### 2.10 Frontier analysis

This study applied frontier analysis to evaluate countries’ efficiency in managing health burden relative to their Socio-demographic Index (SDI) levels. By integrating Global Burden of Disease (GBD) data with SDI metrics, we selected the age-standardized disability-adjusted life years (DALYs) rate as the health burden indicator and constructed a Bootstrap-based data envelopment analysis (DEA) efficiency frontier model (26). This approach identified leading countries and regions, setting benchmarks for others.

## 3 Results

### 3.1 Global trends and temporal dynamics

From 1990 to 2021, the global number of incident cases of varicella and herpes zoster showed a modest increase from 72.83 million (95% UI: 70.11–75.85 million) to 86.68 million (95% UI: 81.69–92.21 million), representing a 19.0% rise. However, the age-standardized incidence rate (ASIR) remained stable (1244.1 vs. 1248.6 per 100,000 population).

Concurrently, global deaths significantly decreased from 15,633 (95% UI: 14,140.8-17,384.6) in 1990 to 13,931 (95% UI: 12,584.9-15,605) in 2021, marking a 10.9% reduction. The age-standardized mortality rate (ASMR) declined by 50.0% (0.4 [95% UI: 0.3–0.4] vs. 0.2 [95% UI: 0.2–0.2] per 100,000). The total disease burden measured in disability-adjusted life years (DALYs) decreased by 16.8%, from 1.065 million (95% UI: 0.937–1.230 million) to 0.886 million (95% UI: 0.744–1.060 million). The age-standardized DALY rate dropped from 19.3 (95% UI: 17.0–22.1) to 12.3 (95% UI: 10.4–14.7) per 100,000 (Table 1).

These findings demonstrate that progressive medical interventions and vaccination programs over the past three decades have effectively reduced mortality and disability impacts, achieving phased successes (4). However, compared with other vaccine-preventable infectious diseases, the global case count of varicella and herpes zoster has increased during this period, particularly with no significant decline in ASIR. The reductions in ASMR and age-standardized DALY rates remain insufficient, indicating that global burden control for varicella and herpes zoster still faces substantial challenges.

### 3.2 Analyse of joinpoint

Our joinpoint regression analysis of global varicella and herpes zoster incidence (1990–2021) revealed heterogeneous trends across regions despite overall decline. The post-2019 acceleration (APC = -12.82) suggests NPIs’ collateral benefits, while High-income North America’s post-2005 reversal (APC = +6.92) reflects declining vaccination adherence (27). North Africa/Middle East’s deterioration (APC = +10.81) correlated with conflict impacts (28), and Southern sub-Saharan Africa’s post-2019 rise (APC = +0.89) highlighted pandemic disruptions in vulnerable regions (29). The Supplementary data of AAPC are presented in Table 1, APC data and inflection point data can be viewed in Annex Excel sheet2.

### 3.3 Age and sex differences and time trends

**Figure 1:**
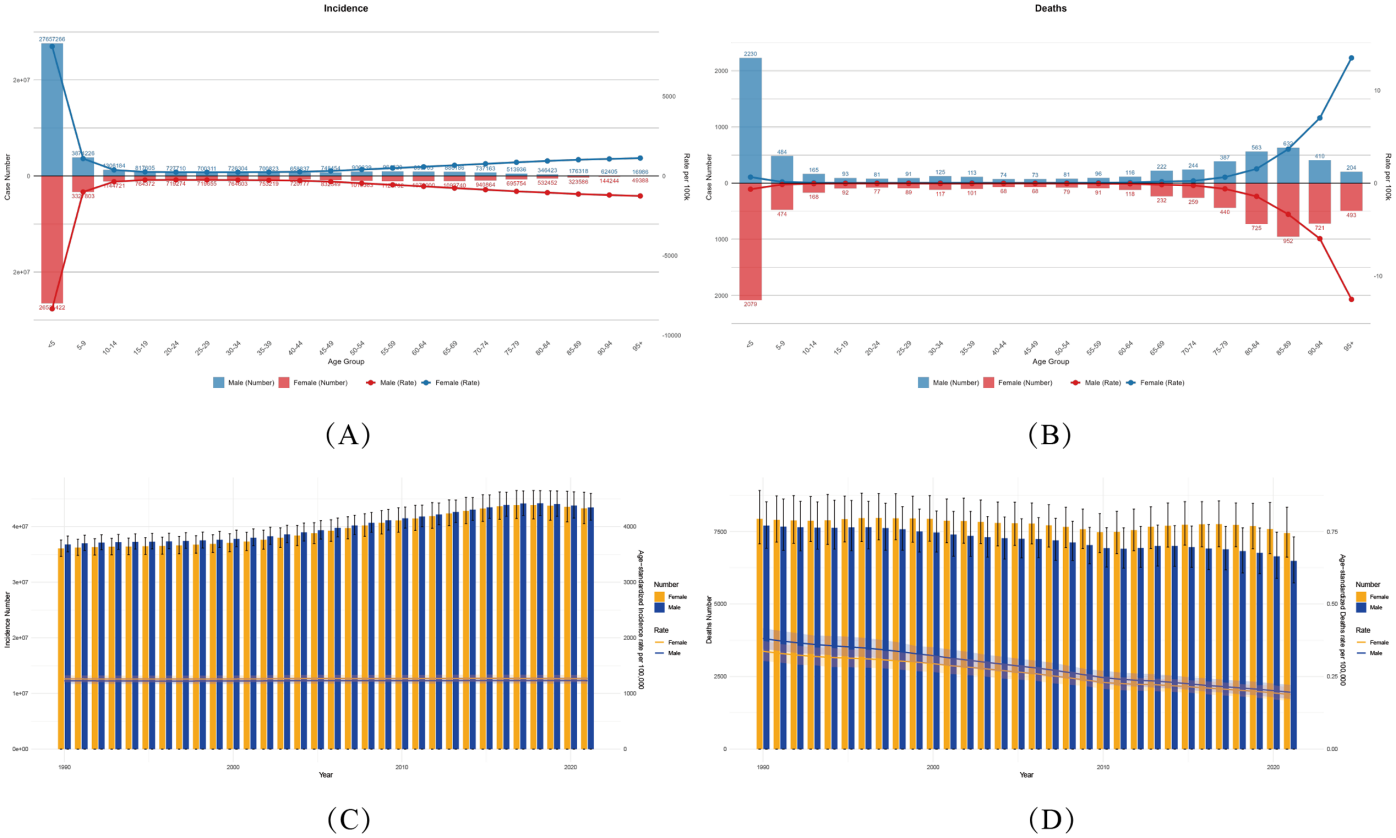
Global varicella and herpes zoster Burden (1990–2021) with Temporal Trends by Sex. **(A,B)** Age-sex stratified incident cases/deaths (2021) with age-standardized rates (ASIR/ASMR). Numbers above bars: case counts. **(C,D)** Temporal trends (1990–2021) in ASIR/ASMR by sex. Bands: 95% UIs for rates; error bars: 95% UIs for counts (ASIR, age-standardized incidence rate; ASMR, age-standardized mortality rate; ASDR, age-standardized DALYs rate).

Age-specific analysis in 2021 revealed a nonlinear burden trajectory of varicella and herpes zoster, demonstrating significant age-dependent patterns globally. Children under five exhibited the highest incidence rates, with 26.52 million cases (95% UI: 23.38–29.18 million) in females and 27.66 million (95% UI: 24.05–30.79 million) in males, accounting for 30.6–31.9% of total cases (Figure 1). This aligns with immature immune systems and heightened susceptibility to chickenpox in early childhood.

A dramatic 97.04% decline in cases occurred among adolescents (15–19 years) compared to the 0–5 age group (females: 764,000; males: 818,000), suggesting vaccine-coverage effects. However, case numbers resurged in adults ≥45 years, peaking in the 55–59 age group (females: 1.12 million; males: 960,000), attributable to immunosenescence and escalating herpes zoster risk. It highlights the age-related complexity of varicella-zoster virus (VZV) infection.

Mortality and disability burden (DALYs) followed a distinct U-shaped age distribution. Under-five males had the highest deaths (2,230; 95% UI: 1,700–2,825), representing 34% of male fatalities, while females in this age group accounted for 28% (2,079 deaths; 95% UI: 1,647–2,575). DALYs were disproportionately concentrated in young children: males under five bore 187,750 DALYs (95% UI: 148,770–232,749), versus 201,205 (95% UI: 153,003–255,253) in females (7.2% difference), constituting 21.2–22.7% of total DALYs.

Older populations showed alarming rises in age-standardized DALY rates (ASDR). From 60–64 years onward, ASDR increased steeply with each 5-year age increment, with males consistently exceeding females (≥60 years). Peak ASDR occurred at age 95 + (males: 120/100,000; 95% UI: 96–146), 2.03-fold higher than the 0–5 group (120.35 vs. 59.18). Notably, higher comorbidity burdens in males exacerbated sex disparities, warranting further investigation.

### 3.4 Gender disparities and dynamic trends

The 2021 global analysis of gender disparities in varicella and herpes zoster disease burden demonstrated consistently higher incidence and mortality rates among males during childhood and older adulthood. For example, males under five years old had 4.3% more cases than females (27.66 million vs. 26.52 million), with a 7.62% higher mortality rate (2,230 vs. 2,079 deaths). In older adults (≥60 years), elevated male mortality may correlate with weaker immune responses and higher comorbidity burdens.

By contrast, the disease burden among women of childbearing age (15–49 years) is slightly higher than that among men. Taking the 30–34 age group as an example, the incidence rate in women is as high as 764,600 cases (95% Confidence Interval: 527,800-1,018,100), compared with 726,300 cases in men (95% Confidence Interval: 496,900-976,700). This difference may be related to immunosuppression during pregnancy. Notably, the difference in mortality rates in this age group is negligible (117 deaths in women vs. 125 deaths in men). This gender difference persists in the 35–39 age group, where the incidence rate in women remains high (753,200 cases vs. 700,800 cases in men).

**Figure 2:**
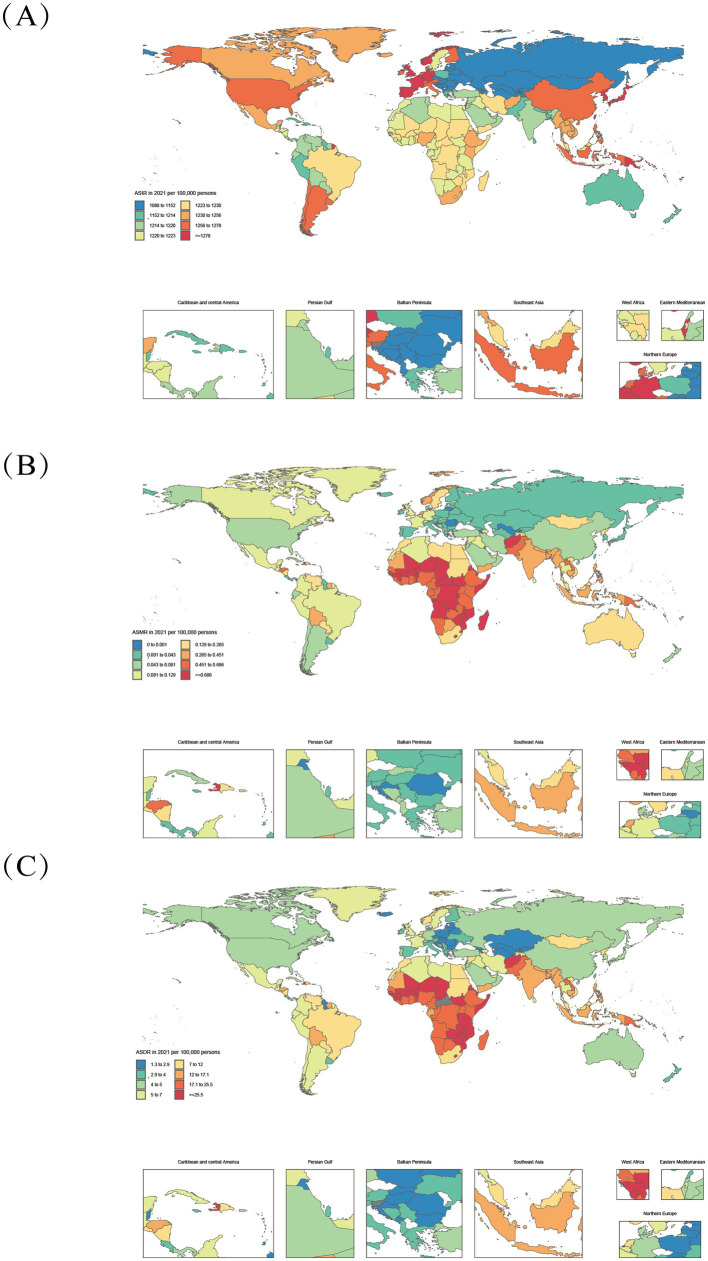
Age-standardized varicella and herpes zoster Burden in 204 Countries (2021). **(A–C)**: ASIR, age-standardized incidence rate; ASMR, age-standardized deaths rate; ASDR, age-standardized DALYs rate.

In the older population aged 70–74 years, the DALYs (Disability-Adjusted Life Years) of women (14,131.01; 95% Confidence Interval: 9,994.44–19,562.38) are 17.8% higher than those of men (11,994.97; 95% Confidence Interval: 8,688.32–16,347.13) (*p* < 0.05) (Figure 2). The burden of varicella and herpes Zoster gradually shifts from a disadvantage in males during infancy to a predominance in older females. This difference provides key evidence for gender- and age-stratified interventions: strengthening immune protection for male infants, monitoring pregnancy-related risks in women of childbearing age, and preventing VZV sequelae in older females.

### 3.5 Gender differences and time trends across all age groups (1990–2021)

Our analysis of global varicella-zoster virus (VZV) cases from 1990 to 2021 revealed gender disparities and temporal trends. In terms of lifetime incidence, males consistently recorded higher cumulative cases than females (43.418 million vs. 43.26 million in 2021). While male annual incidence rates (ASIR, 1,369.5/100,000) slightly exceeded females’ rates (1,361.4/100,000) in 1990, this gap narrowed significantly by 2021 (from 1,096.6/100,000 to 1,100.2/100,000 for males versus females), indicating a more pronounced decline in male incidence (20.0% reduction vs. 19.2% in females). However, the baseline data showed minimal gender differences.

Regarding disability-adjusted life years (DALYs), male lifetime DALYs persistently surpassed female totals from 1990 to 2012, though the disparity gradually diminished. A reversal occurred in 2012, with female DALYs exceeding males thereafter (437,000 in males vs. 449,000 in females in 2021). Age-standardized DALY rates mirrored this trend: male rates (20.15/100,000) significantly exceeded female rates (19.79/100,000) in 1990, but this pattern inverted by 2021 (11.04/100,000 in males vs. 11.42/100,000 in females), particularly as female DALY rate declines slowed after 2015.

The mortality gap has shown significant temporal variation. Women consistently exhibited higher age-standardized mortality rates than men (0.300/100,000 for women versus 0.287/100,000 for men in 1990), with the disparity widening to 0.189/100,000 versus 0.164/100,000 by 2021—a gender gap increase exceeding 10%. This disparity became particularly pronounced among individuals aged 65 and above, likely linked to increased exposure risks due to women’s extended life expectancy. However, overall mortality rates for both genders declined synchronously between 1990 and 2021. These findings underscore the critical need to prioritize vaccination efforts and complication management for older women to mitigate gender health disparities (Figure 1).

### 3.6 Regional heterogeneity and socioeconomic determinants

The global burden of varicella and herpes zoster exhibits significant geographical and socioeconomic gradients across 204 countries and 21 regions, with differential patterns linked to the Socio-demographic Index (SDI) that demonstrate both universal trends and complex variations due to disease-specific characteristics and population vulnerability. From 1990 to 2021, the global age-standardized mortality rate (ASMR) and disability-adjusted life years rate (DALYs_rate) for varicella and herpes zoster declined from 0.35/100,000 and 19.28 to 0.19/100,000 and 12.31, respectively, though with marked regional heterogeneity. High-SDI regions (e.g., Australasia and Western Europe) consistently showed lower disease burdens than low-SDI regions. In 2021, Australasia’s DALYs_rate was merely 4.50, while Central Sub-Saharan Africa reached 25.03—a 5.6-fold disparity, reflecting profound gaps in healthcare accessibility and public health infrastructure. Notably, the burden displays a bimodal distribution: low-SDI regions (SDI < 0.5) are characterized by high ASMR and age-standardized DALYs rate (ASDR), whereas high-SDI regions (SDI > 0.8) are dominated by elevated age-standardized incidence rates (ASIR).

In 2021, low- and low-middle-SDI regions (SDI < 0.4) accounted for 67.2% of global varicella-related deaths (9,369 cases) and 66.9% of varicella-related DALYs (592,474). Persistent high burdens were observed in low-SDI regions like Sub-Saharan Africa (Central African Republic: ASMR 1.85/100,000, DALYs_rate 59.91/100,000) and South Asia (Afghanistan: ASMR 1.20/100,000, DALYs_rate 39.93/100,000) (Figure 1), driven by inadequate vaccine coverage, weak primary healthcare, and high population density facilitating transmission. Despite reductions (e.g., Central Africa’s ASMR declined from 1.38 to 0.76/100,000), the pace lagged behind global averages, with SDI remaining below 0.5 (2021: 0.47), underscoring chronic deficits in vaccination and health services. Although economic growth in some low-SDI regions (e.g., West Africa’s SDI rose from 0.27 to 0.45) reduced ASMR by 40.94%, absolute burdens remained high, confirming that economic progress alone cannot resolve health inequities without addressing geographical and policy factors.

**Figure 3:**
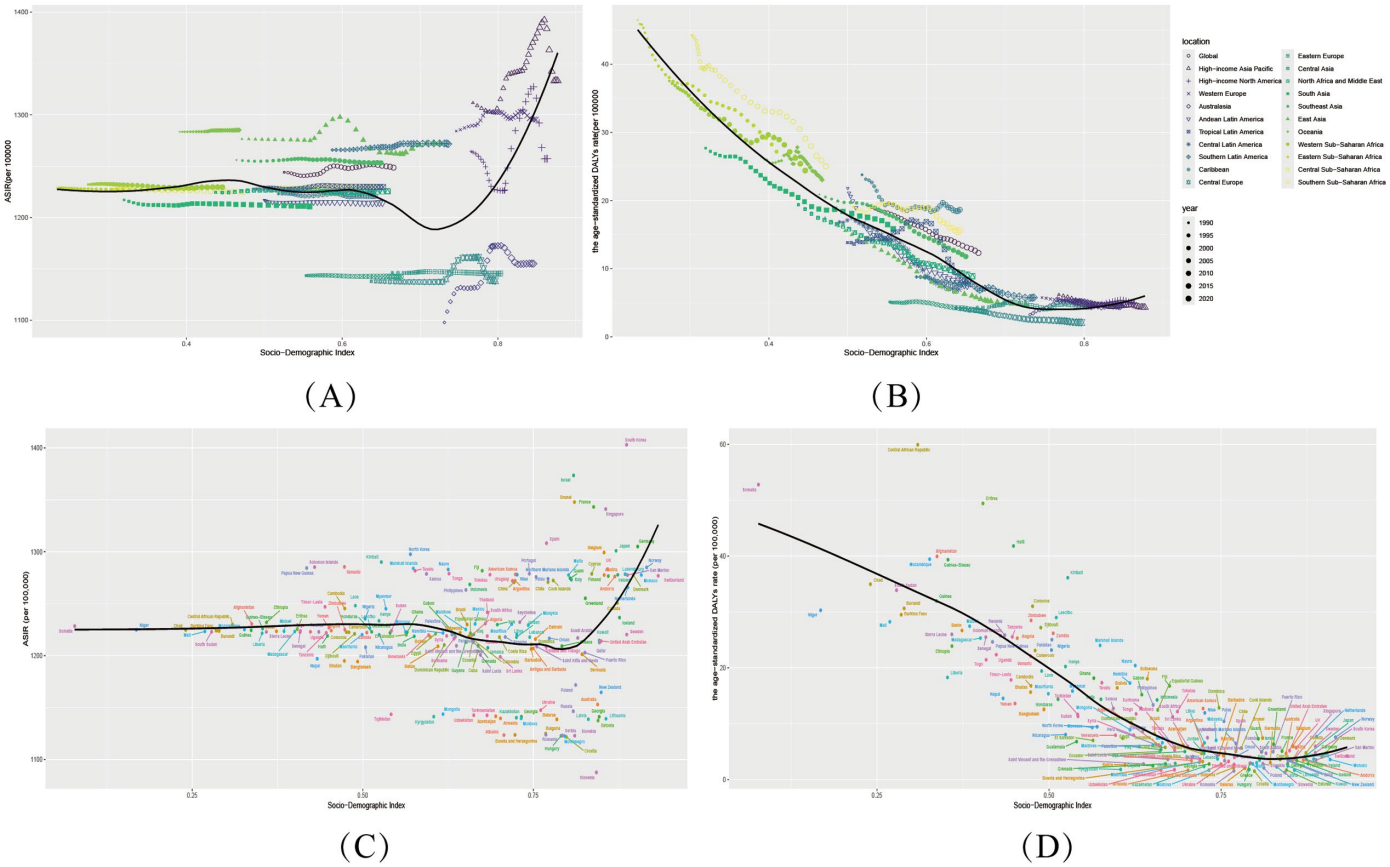
Sociodemographic Index associations with varicella and herpes zoster Burden in 204 Countries and 21 regions. **(A,B)** Age-standardized incidence (ASIR) and DALY rates (ASDR) across 21 regions by SDI (1990–2021). **(C,D)** Country-level ASIR and ASDR versus SDI (2021). Shaded areas/error bars represent 95% uncertainty intervals.

Middle- and high-SDI regions exhibited divergent trajectories: Latin America (SDI 0.49 → 0.64) achieved a 65.3% ASMR decline, while East Asia (SDI 0.47 → 0.73) leveraged mass immunization to slash ASMR by 88%, exemplifying inverse SDI-burden correlations. Conversely, high-SDI regions like Australasia saw ASMR rise from 0.07 to 0.14 (1990–2000), and North America’s herpes zoster ASIR remained elevated (1990: 1264.25/100,000; 2021: 1257.32/100,000), signaling aging-driven epidemiological shifts. A threshold effect emerged: when SDI exceeded 0.6 (e.g., Central Asia: SDI 0.66 → 0.68), ASMR reductions slowed (2015–2021: only 22%) (Figure 3), indicating diminishing marginal returns. In high-SDI nations (SDI > 0.8), varicella neared elimination (ASMR<0.1/100,000, DALYs_rate<5/100,000) due to >90% vaccine coverage (e.g., Norway, Japan), but herpes zoster ASIR surged (e.g., Japan: 1300/100,000 vs. global 1248/100,000) amid aging populations (28.8% aged ≥65) (30), highlighting”aging-associated disease resurgence” as a public health challenge.

Critically, SDI-burden associations follow nonlinear thresholds: Low-SDI phase (SDI < 0.5): Each 0.1-unit SDI increase reduced varicella ASMR by ~30% (e.g., Niger SDI 0.17 vs. Bangladesh SDI 0.49: ASMR 0.95 → 0.30/100,000), attributable to improved basic healthcare and nutrition. Middle-SDI phase (0.5–0.7): Heterogeneity widened—e.g., Bolivia (SDI 0.60, ASMR 0.42/100,000) and Syria (SDI 0.62, ASMR 0.11/100,000) showed 3-fold differences, emphasizing vaccination policies’ pivotal role. High-SDI phase (>0.8): Marginal gains diminished; herpes zoster replaced varicella as the focus (e.g., South Korea: ASIR 1403.0/100,000).

Outliers revealed confounding factors: Somalia (SDI 0.31, ASMR 1.80/100,000), with 70% healthcare infrastructure destroyed, became a high-burden outlier, while Bhutan (SDI 0.47, ASMR 0.37/100,000) and Nepal (SDI 0.43, ASMR 0.38/100,000) likely benefited from cultural isolation and herbal medicine. High-SDI outliers like Israel (ASIR = 1373.5/100,000) and South Korea (ASIR = 1403.0/100,000) reflected diagnostic intensity (PCR testing) (Figure 3). These deviations underscore that beyond SDI, vaccination policies, cultural practices, and aging structures are critical intervention targets.

### 3.7 Decomposition analysis

**Figure 4:**
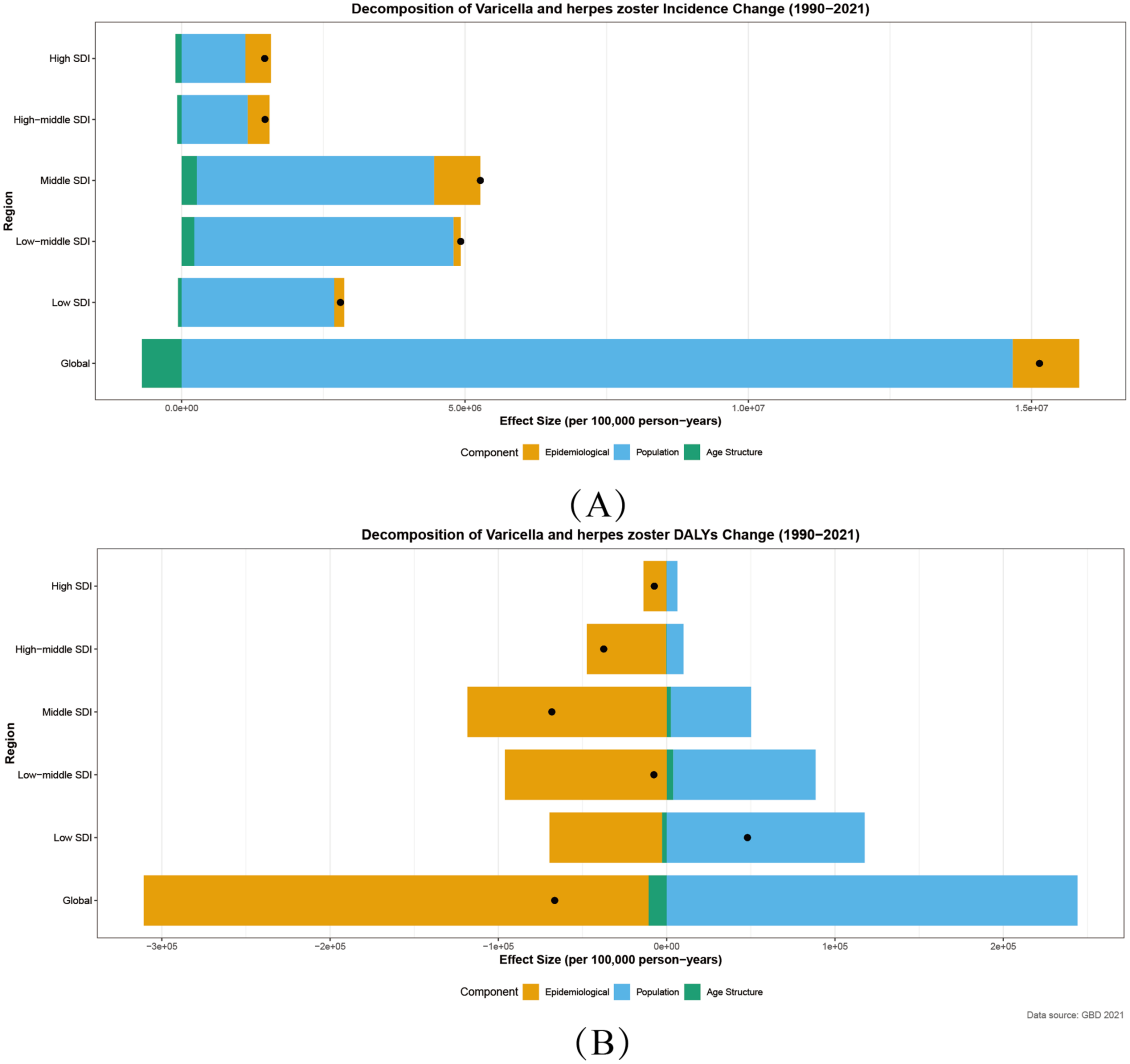
Decomposition of Incidence **(A)** and DALYs **(B)** for varicella and herpes zoster by SDI, 1990–2021. Age Structure:a_effect: aging population structure effect; Population:p_effect: population growth effect; Epidemiology:r_effect: incidence rate effect; Black Point:total_diff: total difference.

Leveraging the 2021 Global Burden of Disease (GBD) data, this study employed Socio-demographic Index (SDI)-based decomposition analysis to reveal significant regional heterogeneity in age-standardized incidence rates (ASIR) and age-standardized DALYs rates (ASDR) for varicella and herpes zoster. Globally, incident cases increased by 13,847,350 from 1990 to 2021, with a total decomposition effect (total_diff) of 15.13 million. Population growth drove 95.8% of this rise (p_effect = 14.66 million), while aging demographics exerted a protective effect (a_effect = −0.70 million). (Figure 4).

### 3.8 Incidence decomposition by SDI region

ASIR trends followed an “intermediate-high, polar-low” distribution:

middle SDI regions showed the highest absolute increase (total_diff = 520,000), fueled by combined population growth (p_effect = 4.18 million) and epidemiological escalation (r_effect = 810,000), likely reflecting inadequate vaccination coverage to match demographic pressures. High SDI regions exhibited a “high-intervention, high-rebound” paradox: despite population-driven contributions (p_effect = 1.12 million), epidemiological rates rose counterintuitively (r_effect = 450,000, Figure 4).

### 3.9 DALYs decomposition patterns

Global DALYs declined (total_diff = −67,000), primarily due to reduced incidence (r_effect = −300,000), though population growth (p_effect = 240,000) offset 41.7% of gains. Regional disparities were stark: low SDI regions experienced DALYs increases (total_diff = 48,000), trapped in a vicious cycle of population growth (p_effect = 120,000) and healthcare deficits. Middle SDI regions achieved the steepest declines (total_diff = −68,000), with epidemiological improvements (r_effect = −120,000) contributing 173.5% of the total effect (Figure 4).

These patterns underscore the critical role of equitable vaccine delivery and healthcare strengthening to mitigate demographic pressures, particularly in resource-limited contexts. The analysis delineates how varicella and herpes zoster burden dynamics are shaped by intersecting demographic, epidemiological, and healthcare access factors across the SDI spectrum.

### 3.10 Bayesian age-period-cohort (BAPC) modeling and sex-specific incidence projections to 2036

Our BAPC modeling of global varicella and herpes zoster age-standardized incidence rates (ASIR per 100,000) reveals an overall declining trend from 1990 to 2036, with gradually narrowing sex disparities. Historical data (1990–2021) shows that the difference in incidence rates between men and women is very small.

Projections to 2036 indicate female ASIR will further decline to 1003.72 (95% UI: 827.54–1176.25) and male ASIR to 992.35 (822.27–1156.24), representing reductions of 8.8 and 9.5% from 2021 levels, respectively. Notably, females projected to show 1.1% higher incidence than males (1003.72 vs. 992.35). Uncertainty intervals widen substantially over time (female UI range: ±71.30 in 2021 to ±174.36 in 2036; male: ±60.77 to ±166.99). Despite this, stable annual decline rates (females: 0.35%; males: 0.40%) suggest sustained public health intervention efficacy.

### 3.11 Sex-specific dynamics and implications

**Figure 5:**
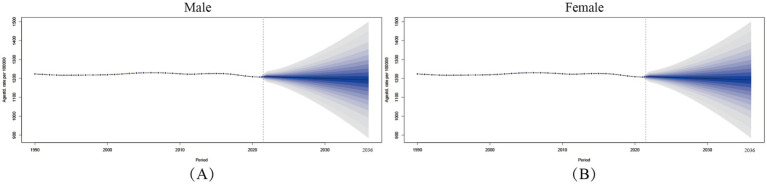
Varicella-Zoster Incidence Projections. ASIR rate projections of varicella and herpes zoster among male **(A)** and female **(B)** in the Global population, 1990–2036.

Diverging trends highlight the need for targeted prevention: Females show slower ASIR declines during reproductive ages (20–40 years: 0.28% vs. 0.33% annually in males), possibly due to pregnancy-related immunosuppression. Males exhibit greater reductions in older adults herpes zoster incidence (11.2% decline by 2036 vs. 2021) (Figure 5).

### 3.12 Frontier analysis

Our frontier analysis highlights striking disparities between countries in 2021. Notably, 10 nations—including the Central African Republic, Haiti, and Eritrea—exhibited markedly higher age-standardized burden rates, placing them far from the optimal frontier. This indicates substantially worse outcomes than expected given their development levels.

Among low-SDI countries (SDI < 0.5), Somalia, Niger, Mali, Nepal, and Liberia demonstrated elevated absolute burdens. However, when accounting for their baseline SDI, these nations clustered closer to the frontier, suggesting their outcomes align with constrained-resource expectations.

**Figure 6:**
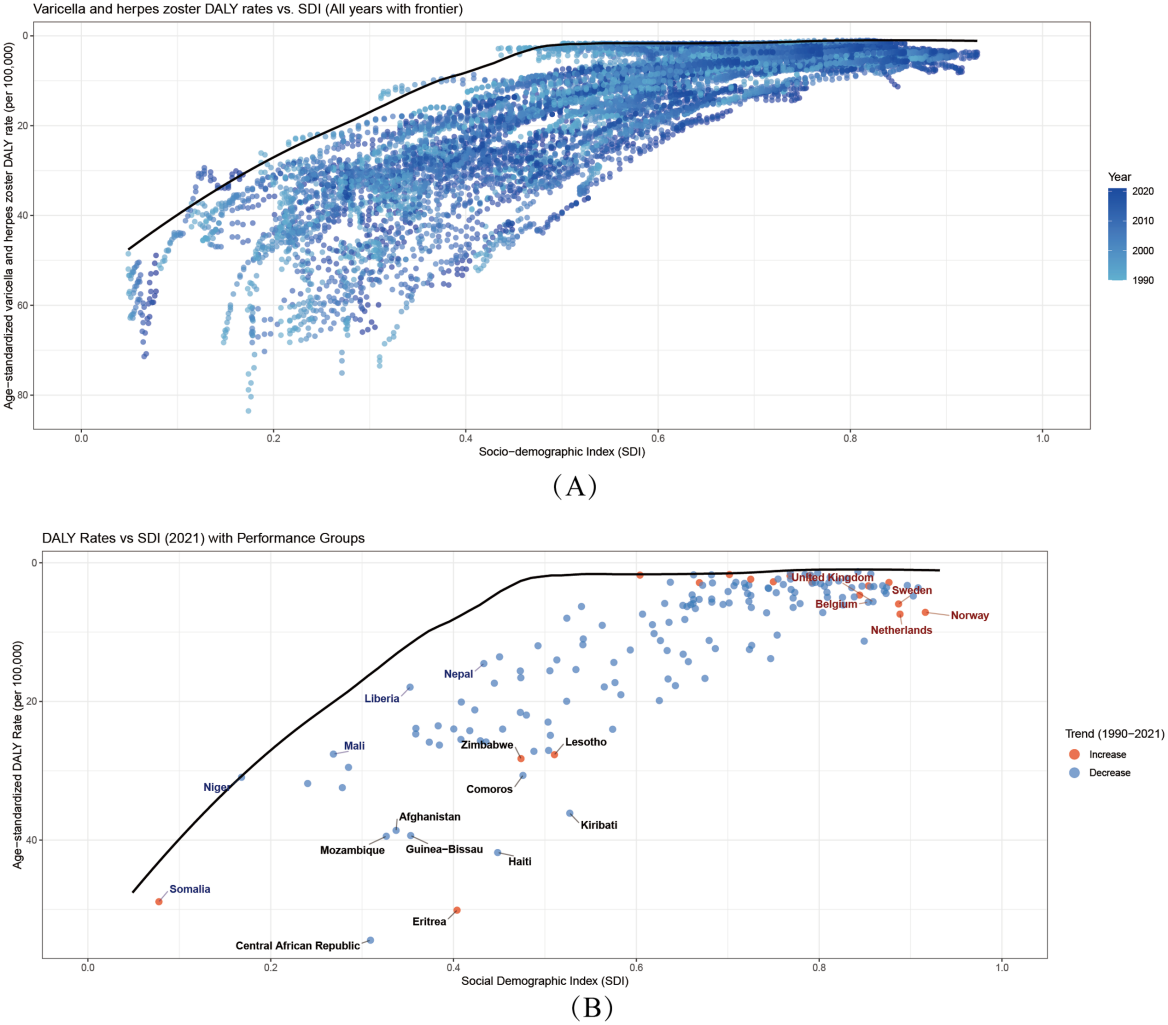
Global Burden-SDI Relationships and Frontier analysis. **(A)** Age-standardized varicella and herpes zoster DALY rates versus SDI (1990–2021). Points colored by year. Black line shows optimal performance frontier. Higher vertical positions indicate worse outcomes. SDI ranges 0–1 (low-high development). **(B)** 2021 data highlighting trends (blue = 1990–2021 decrease; red = increase). Annotated extremes: Blue: 5 best-performing low-SDI countries Red: 5 worst-performing high-SDI countries Black: 10 countries furthest from frontier. Frontier represents minimum achievable burden at each development level.

Conversely, high-SDI countries (SDI > 0.85) like Belgium, the United Kingdom, and Norway showed significant effective gaps versus their development potential. Their DALYs rates fell substantially farther from the ideal frontier than anticipated—a surprising finding that underscores how even advanced health systems may be underperforming in addressing varicella and herpes zoster burdens (Figure 6).

## 4 Discussion

### 4.1 A dual-disease paradigm tracking global development

VZV causes a unique dual-disease paradigm, exhibiting distinct epidemiological patterns that closely track societal development. Varicella-zoster virus (VZV) infection typically manifests as varicella (chickenpox) in children, whereas in immunocompromised adults, particularly the older adults, it is more likely to present as herpes zoster (shingles) (31). This age-bimodal manifestation creates complex public health challenges: low-SDI regions struggle with high childhood varicella incidence due to limited vaccine access, while high-SDI nations face growing zoster burdens from population aging. Despite WHO recommendations, global varicella vaccine coverage remains uneven. Notably, unlike routine vaccines such as measles, VZV vaccination remains excluded from many national immunization programs. This study confirms that regional disease burden disparities primarily arise from the interplay of Socio-demographic Index (SDI), population structure, and policy effectiveness, with healthcare performance, vaccine access, and demographic transitions as key determinants.

### 4.2 Global progress, persistent disparities, and key determinants

Analysis of global data from 1990 to 2021 reveals a complex epidemiological picture. While absolute case numbers demonstrated an upward trajectory, age-standardized incidence rates remained stable, accompanied by significant reductions in both mortality and disability-adjusted life years (DALYs). These positive trends represent a major achievement attributable to three key technological advancements: (1) Diagnostic Advancements, including PCR-based detection and direct fluorescent antibody testing (31), enabling early pathogen identification; (2) Therapeutic Innovations, such as acyclovir, valacyclovir, and brivudine (32), which reduce disease duration and severity (33); and (3) Vaccination Progress, with multinational reviews confirming >80% reductions in varicella incidence and hospitalization following vaccine introduction (34, 35).

However, the stable age-standardized rate alongside a rising number of absolute cases indicates that global population growth and extended life expectancy have significantly increased the varicella-zoster virus (VZV)-related burden (36), a high-risk group for VZV reactivation due to immunosenescence (37). This phenomenon drives the profound regional disparities observed. The burden of VZV exhibits a characteristic bimodal distribution: low-SDI regions (SDI < 0.5) show high age-standardized mortality and DALY rates, while high-SDI regions (SDI > 0.8) are dominated by elevated incidence rates. Vaccination coverage is pivotal (38); the U.S. program, for example, led to a 98.6% decline in varicella incidence (39). In contrast, Norway’s lack of routine vaccination resulted in annual healthcare costs of €9 million (40).

The aging of populations in high-SDI regions presents a new frontier of challenges. By 2020, adults ≥65 years comprised 29% of Japan’s population, paralleling a 54.5% increase in herpes zoster cases (41). This demographic characteristic helps explain a key finding in our cutting-edge analysis: “efficiency gaps” also exist in high-SDI countries (Figure 6). Some high-SDI countries face severe population aging challenges, and their higher DALY burden does not reflect healthcare system inefficiency but stems from the triple impacts of immunosenescence, aging demographics, and inadequate adult vaccination strategies (42, 43).

Conversely, low-SDI regions face compounded challenges. In Senegal, 44.3% of measles-like cases were VZV-positive (44), and HIV co-infection elevates zoster risk 12-17-fold in Africa. These disparities are exacerbated by inadequate surveillance, cold chain limitations (45), climate factors, and conflict (46), demanding tailored strategies aligned with regional SDI contexts.

Underpinning these trends are pivotal biological determinants. Age dictates a U-shaped burden curve: children under 5 exhibit the highest incidence due to immature immune systems (47, 48), while a resurgence peaks at 55–59 years as age-associated immunosenescence elevates herpes zoster risk (49), with lifetime risk reaching 50% by age 85 (37). Sex-specific variations are also pronounced. Males show higher incidence and mortality in childhood and older adulthood, potentially due to weaker innate immune responses (50) and a higher prevalence of VZV-exacerbated cardiovascular complications (51, 52). Reproductive-age females (15–49 years) bear slightly greater burdens, linked to pregnancy-associated immune modulation and hormonal influences (53–55).

By integrating more demographic indicators (e.g., proportion of population over 65), future applications of this model can enable more comprehensive assessments, thereby measuring healthcare system performance more equitably across different baseline risk contexts. This requires dynamic parameter adjustments based on real-world monitoring data.

### 4.3 The impact of the COVID-19 pandemic: a catalyst for new risks

The COVID-19 pandemic introduced new complexities. Emerging evidence highlights a potential association between COVID-19 and VZV reactivation (32). One retrospective study showed COVID-19 patients had a significantly higher risk of HZ onset within a year (56). However, the relationship is not straightforward, with conflicting data on whether COVID-19 vaccination elevates HZ risk (57, 58), necessitating further research.

The host’s internal environment is a critical variable, with the gut microbiome increasingly recognized as a key regulator of systemic immunity that influences vaccine efficacy and viral immunity, especially in older adults (59). This is exemplified by research from Fu et al. (60), which showed how the maternal gut microbiome and its metabolites influence SARS-CoV-2 vaccine efficacy and mother-to-infant antibody transfer (61). This highlights that host-intrinsic factors are key regulators of vaccine immunogenicity and may explain interindividual variability in viral reactivation risk. This concept is particularly relevant for latent viruses, as emerging research suggests the gut microbiota can influence the balance of herpesvirus immunity and reactivation (62). Therefore, future VZV research must investigate how intrinsic host factors, like the gut microbiome, act as critical modulators of vaccine immunogenicity and the variable risk of viral reactivation.

Beyond direct biological interactions, the pandemic severely disrupted global immunization programs. WHO reported substantial declines in routine vaccination coverage, with 18.1 million children missing their first DTP dose in 2021 (63). Varicella vaccination likely faced parallel drops, as suggested by a 63.1% reduction in MMR vaccinations in the U.S. during early 2020 (64). Our study conducted a Joinpoint analysis on the specific six-year period from 2016 to 2021, which encompasses the three years preceding and following the onset of the pandemic. By allowing for a single turning point in the model, we observed a clear finding: for regions impacted by the pandemic, including Global, High-SDI, Low-SDI, Australia, and North Africa and the Middle East, the number of incident cases of both varicella and herpes zoster markedly increased after 2019 compared to the pre-pandemic period (Supplementary 2). It was evident that 2019, the year of the major outbreak, was the inflection point driving this change. This demonstrates that 2019 was the clear turning point driving this change. Although large-scale outbreaks remain undocumented, localized surges in places like India signal heightened transmission risks (65).

### 4.4 Challenges in varicella and herpes zoster control in vaccine hesitancy

Long-term control faces specific hurdles. While antiviral agents like acyclovir are available, VZV’s ability to establish latency complicates management (66). The therapeutic pipeline shows promise with novel agents (67), but continued development is crucial. Compounding this is the growing phenomenon of vaccine hesitancy, a psychological state of uncertainty regarding immunization (68) that has fueled resurgences of diseases like measles (69). Determinants vary by context: misinformation dominates in high-income countries, while access and cultural barriers are primary in low- and middle-income settings (60). The COVID-19 pandemic has exacerbated this by spawning generalized vaccine skepticism (70). Successful interventions in Denmark, Italy, and France show that targeted campaigns and policies can work (71). For VZV, overcoming this requires enhancing vaccine equity, developing next-generation vaccines, and implementing robust public education.

### 4.5 Future projections and policy recommendations

BAPC modeling projects a declining trend in age-standardized incidence rates for both sexes through 2036, suggesting sustained benefits from current interventions. However, these projections demand regionally stratified policy approaches.

For low-SDI countries (e.g., Yemen), priorities must be strengthening primary healthcare via mechanisms like Gavi, implementing technologies like the controlled temperature chain (CTC), and ensuring vaccination completion through community engagement. Middle-SDI nations require real-time surveillance to detect epidemiological transitions and adaptive vaccination strategies. High-SDI countries must confront aging by including RZV in national formularies, promoting two-dose regimens for the immunocompromised (72), and serving as sentinel sites for VZV strain surveillance. Achieving equitable control requires SDI-phase-specific roadmaps—from basic immunization in fragile states to geriatric-focused prevention in aging societies.

### 4.6 Study limitations and future research directions

While this study provides comprehensive analysis of varicella-zoster virus (VZV) burden, several limitations warrant consideration. A primary limitation is that it is based on GBD estimates, which, while robust for assessing population-level trends, cannot establish causal relationships. For instance, we observed trends in varicella and herpes zoster incidence but cannot definitively attribute these changes solely to vaccination programs, clinical management changes, or other public health interventions, as the data are influenced by numerous confounders, including changes in healthcare access, diagnostic practices, and reporting systems. Further limitations include challenges with GBD data availability, completeness, and accuracy in low-SDI regions, and the potential for the GBD modeling approach to overlook early epidemic signals for rare diseases in high-income settings. Second, our analysis insufficiently incorporates the long-term impacts of COVID-19 pandemic disruptions to vaccination systems, potentially affecting prediction validity. Third, the burden estimates may not fully account for healthcare quality variations and socioeconomic determinants across regions. Fourth, the study lacks granular analysis of viral strain variations and their epidemiological implications due to limited genotype-specific and serological data. Therefore, our findings should be interpreted as highlighting epidemiological patterns that warrant further investigation, rather than serving as a direct basis for specific vaccination policy or clinical management guidelines. Such decisions require dedicated local data and targeted cost-effectiveness studies.

Future research should prioritize: (1) longitudinal assessment of pandemic-related immunization delays using real-world vaccination records; (2) integration of molecular epidemiology to elucidate strain-specific disease patterns; (3) enhanced burden estimation models incorporating healthcare access metrics and deprivation indices; and (4) targeted investigations into vaccine equity barriers, including cold chain optimization in resource-limited settings, improved zoster vaccine coverage among older populations. Addressing these gaps will enable more precise risk stratification and policy formulation for VZV control across diverse epidemiological contexts.

## 5 Conclusion

Over the past three decades, global efforts to prevent and control varicella and herpes zoster have achieved some progress, but the widening socioeconomic gap has undermined these gains, creating a major challenge in the global health field.

On one hand, in resource-scarce regions with low Socio-Demographic Index (SDI), preventable childhood varicella still accounts for most global deaths due to unequal access to vaccines and weak health systems. This highlights the unmet status of global public health goals, making it urgent to advance socioeconomic equity and strengthen primary healthcare.

On the other hand, while high-SDI countries have made public health achievements such as increased life expectancy and successful childhood immunization, they have instead seen a rise in the incidence of herpes zoster among the older adults. As immunized populations age, their cellular immune function declines, reactivating the latent VZV (Varicella-Zoster Virus). Therefore, it is necessary to establish a life-course immunization model.

Age/gender vulnerabilities and population growth have led to an increase in the absolute number of confirmed cases, and the COVID-19 pandemic has disrupted immunization programs, further raising the risk of VZV outbreak resurgence. To achieve the World Health Organization (WHO)’s 2030 targets, an integrated strategic framework is required. Firstly, in low-SDI regions, an equity-oriented vaccination program should be implemented, drawing on the model of the COVID-19 Vaccines Global Access (COVAX) mechanism. Innovative technologies such as mobile cold chain systems should be used to ensure children receive timely vaccinations. Secondly, high-SDI countries need to build age-friendly healthcare systems, incorporate zoster vaccines into national health plans, and establish sound adult immunization registration systems. Finally, a global protection framework should be established, including virus strain surveillance networks, investment in the development of next-generation vaccines, and intervention measures tailored to the gender-specific needs of men and women at different life stages.

In conclusion, preventing and controlling lifelong pathogens like VZV requires the combination of technological innovation and social equity. To address this major public health challenge, global solidarity is of great significance for the future prevention and control of varicella and herpes zoster outbreaks and the development of epidemiological immunization strategies.

## Data Availability

The original contributions presented in the study are included in the article/Supplementary material, further inquiries can be directed to the corresponding author.

## Footnotes

1
https://vizhub.healthdata.org/gbd-result/

2
http://ghdx.healthdata.org/

3
https://www.who.int/gho

4
https://www.healthdata.org/Data-tools-practices/data-practices/data-transparency
