# Supplementary figures and images for: Global burden of varicella and herpes zoster across 204 countries, 1990–2021: a temporal trend analysis in the era of the COVID-19 pandemic and projections to 2036
Source: Front Public Health. 2025 Dec 5;13:1654535. doi: 10.3389/fpubh.2025.1654535 (PMC12714960; doi:10.3389/fpubh.2025.1654535)

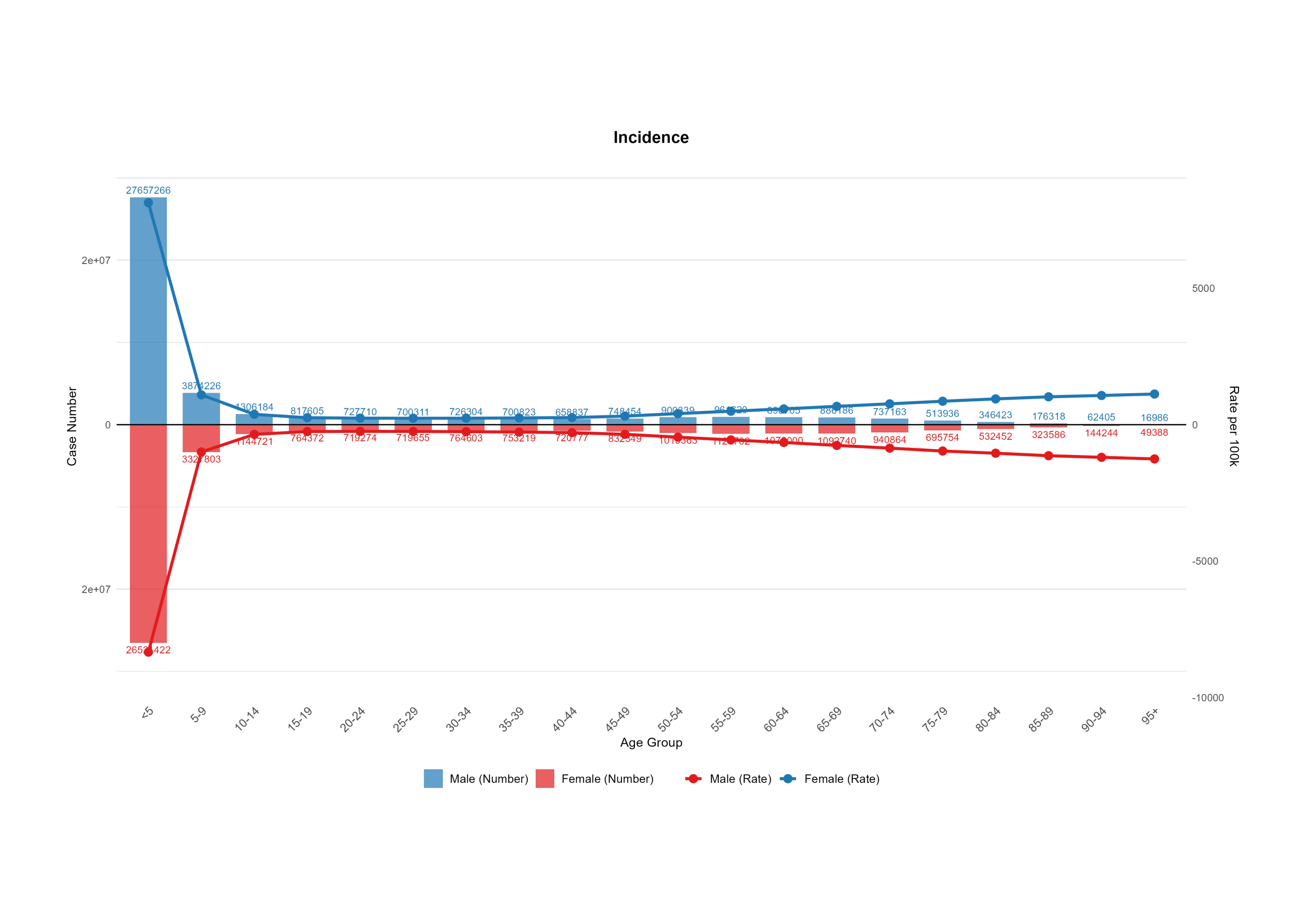

Supplement: Supplementary file 3 [file Image_1.tiff]

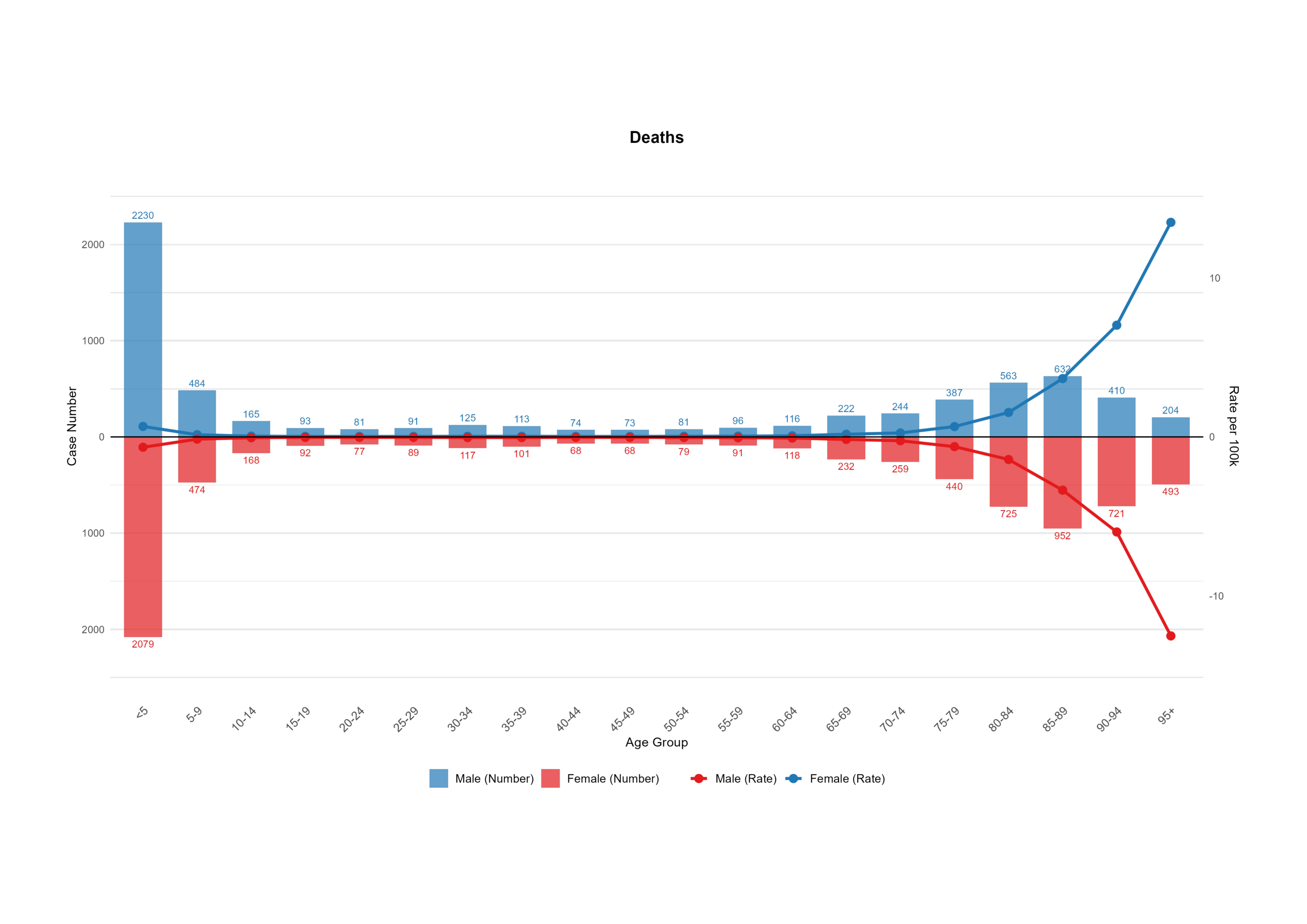

Supplement: Supplementary file 4 [file Image_2.tiff]

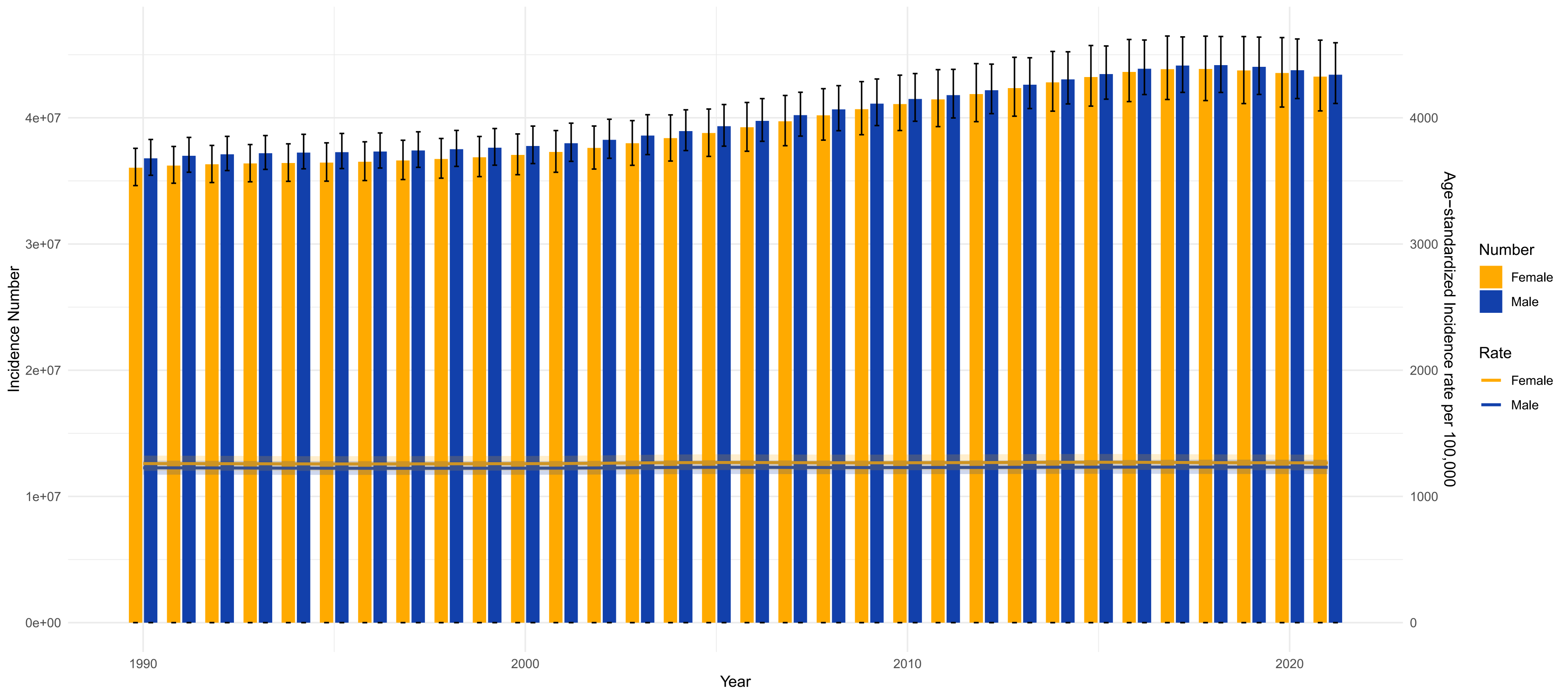

Supplement: Supplementary file 5 [file Image_3.tiff]

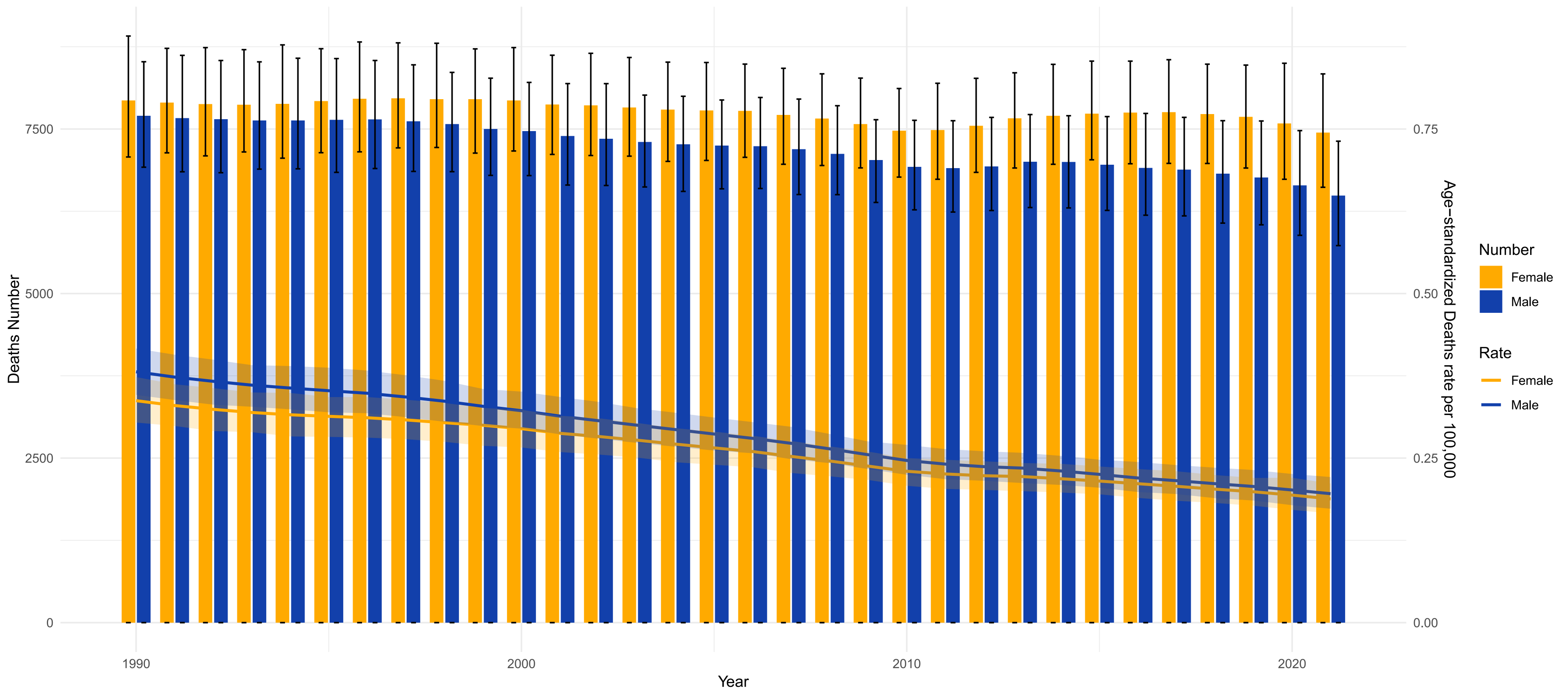

Supplement: Supplementary file 6 [file Image_4.tiff]

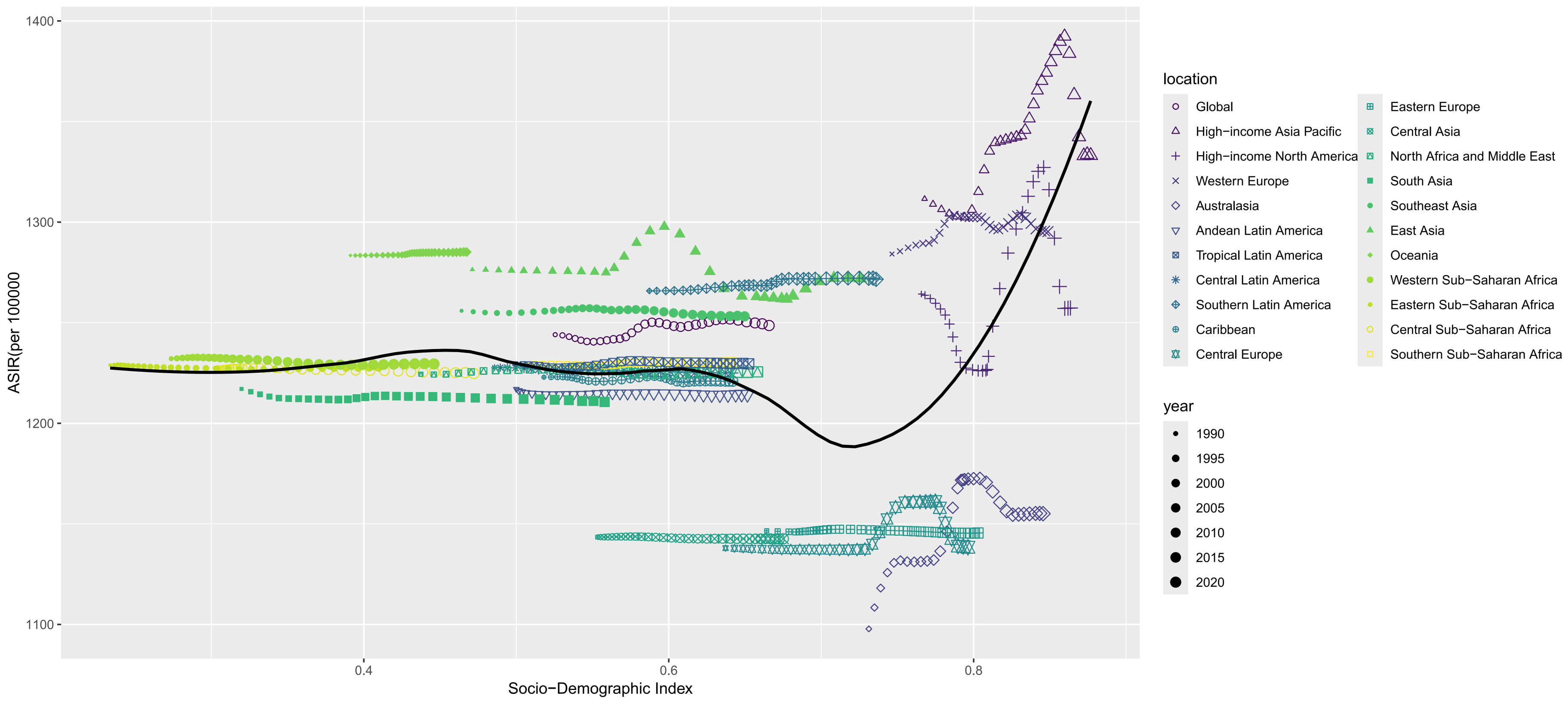

Supplement: Supplementary file 7 [file Image_5.tiff]

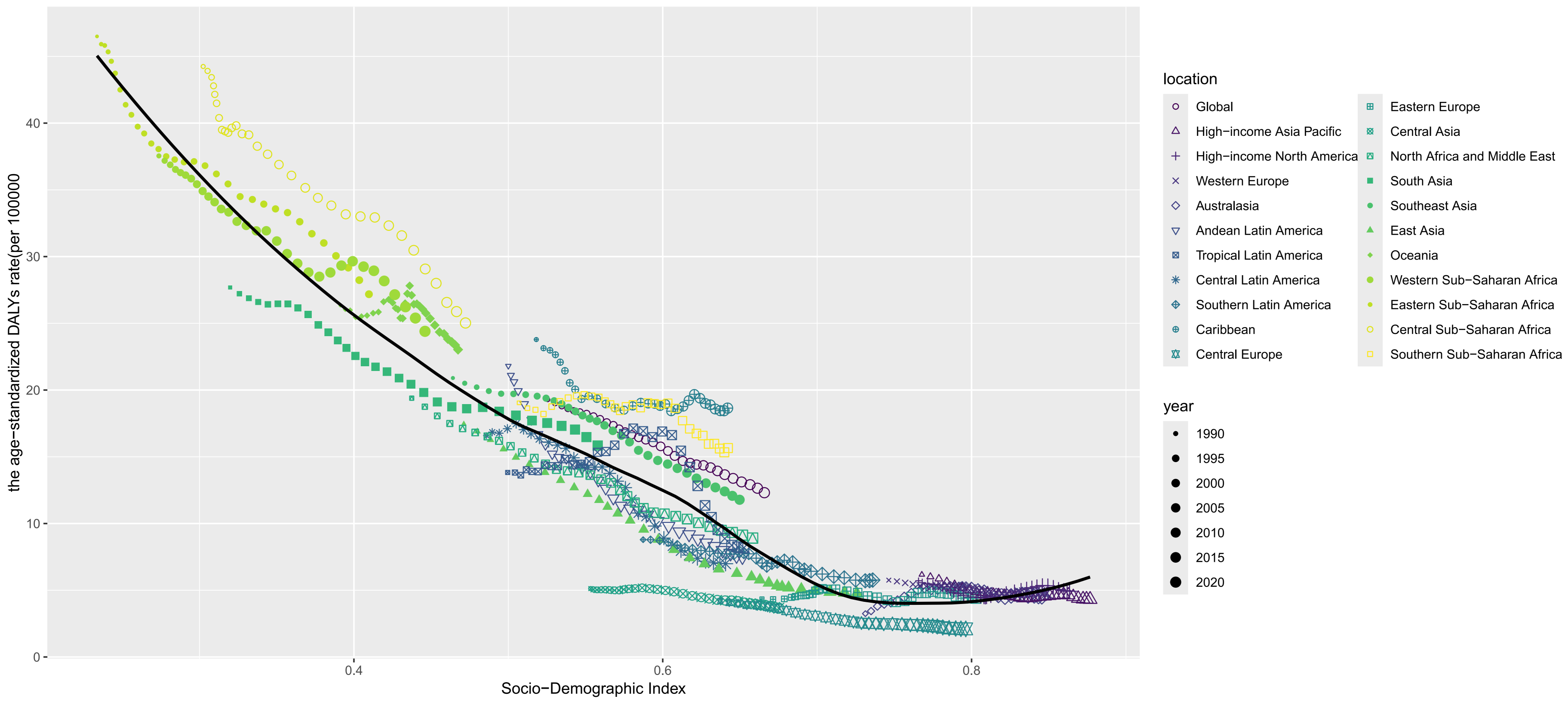

Supplement: Supplementary file 8 [file Image_6.tiff]

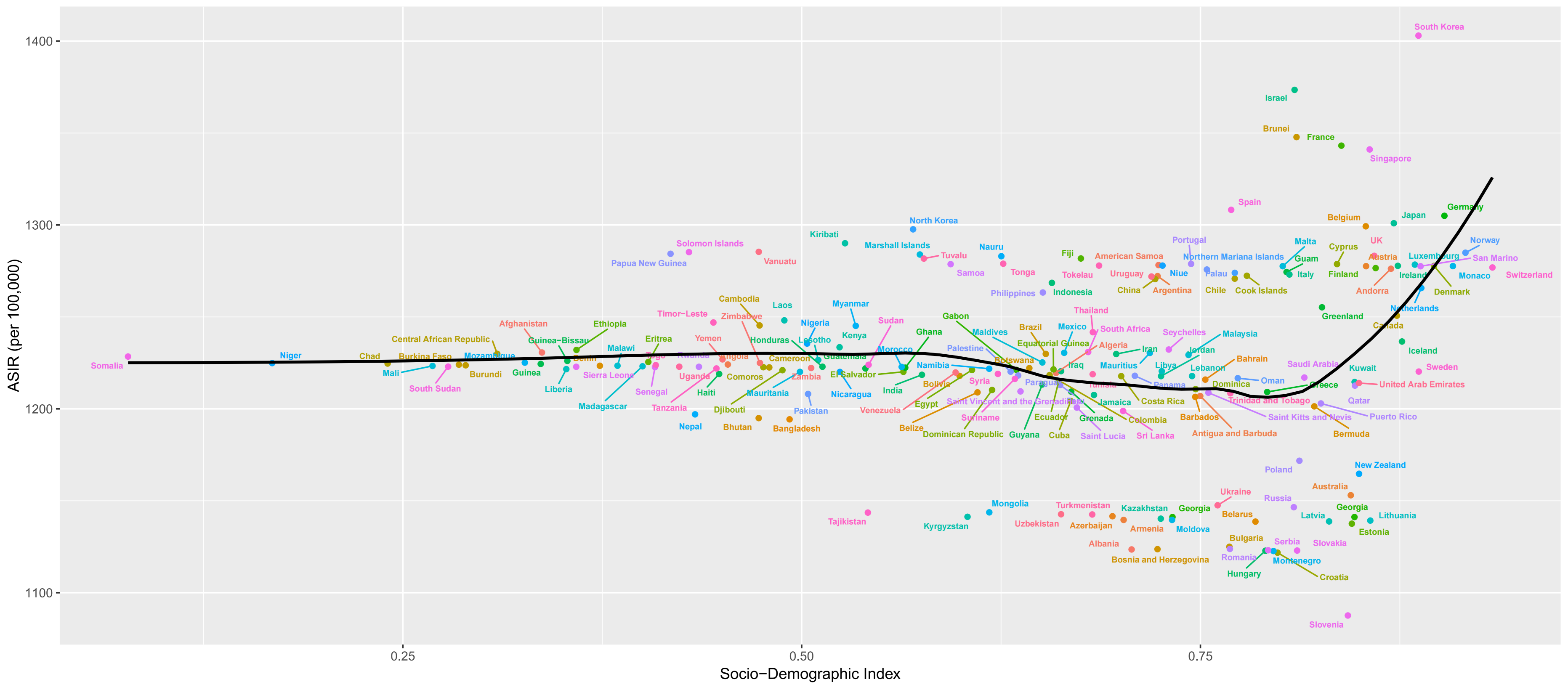

Supplement: Supplementary file 9 [file Image_7.tiff]

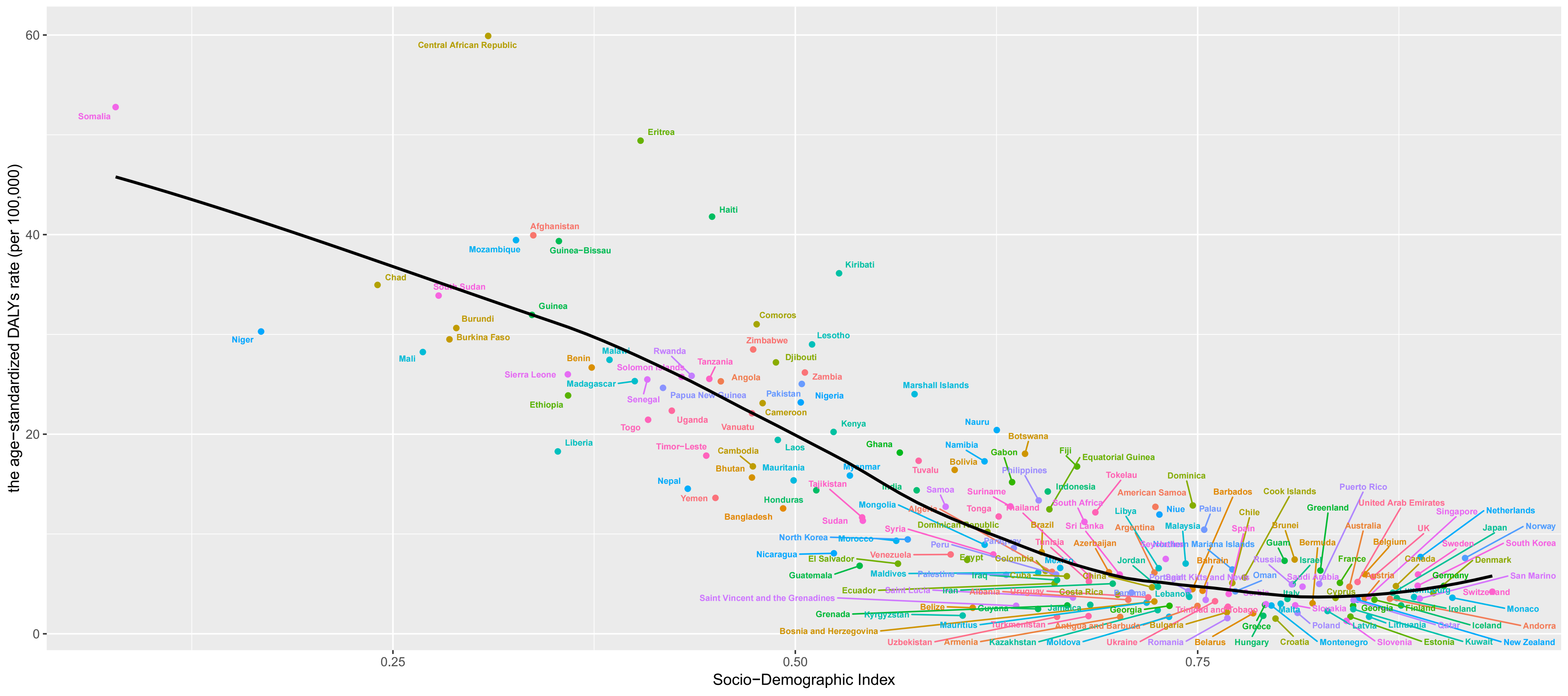

Supplement: Supplementary file 10 [file Image_8.tiff]
